# Stratifying risk of disease in haematuria patients using machine learning techniques to improve diagnostics

**DOI:** 10.3389/fonc.2024.1401071

**Published:** 2024-05-08

**Authors:** Anna Drożdż, Brian Duggan, Mark W. Ruddock, Cherith N. Reid, Mary Jo Kurth, Joanne Watt, Allister Irvine, John Lamont, Peter Fitzgerald, Declan O’Rourke, David Curry, Mark Evans, Ruth Boyd, Jose Sousa

**Affiliations:** ^1^ Personal Health Data Science Group, Sano – Centre for Computational Personalised Medicine - International Research Foundation, Krakow, Poland; ^2^ South Eastern Health and Social Care Trust, Ulster Hospital Dundonald, Belfast, United Kingdom; ^3^ Clinical Studies Group, Randox Laboratories Ltd., Co., Antrim, United Kingdom; ^4^ Belfast Health and Social Care Trust, Belfast City Hospital, Belfast, United Kingdom; ^5^ Northern Ireland Clinical Trials Network, Belfast City Hospital, Belfast, United Kingdom; ^6^ Centre for Public Health, Institute of Clinical Sciences, Queen’s University, Belfast, United Kingdom

**Keywords:** biomarkers, bladder cancer, haematuria, machine learning, stratification, decision support system, unbalanced data

## Abstract

**Background:**

Detailed and invasive clinical investigations are required to identify the causes of haematuria. Highly unbalanced patient population (predominantly male) and a wide range of potential causes make the ability to correctly classify patients and identify patient-specific biomarkers a major challenge. Studies have shown that it is possible to improve the diagnosis using multi-marker analysis, even in unbalanced datasets, by applying advanced analytical methods. Here, we applied several machine learning algorithms to classify patients from the haematuria patient cohort (HaBio) by analysing multiple biomarkers and to identify the most relevant ones.

**Materials and methods:**

We applied several classification and feature selection methods (k-means clustering, decision trees, random forest with LIME explainer and CACTUS algorithm) to stratify patients into two groups: healthy (with no clear cause of haematuria) or sick (with an identified cause of haematuria e.g., bladder cancer, or infection). The classification performance of the models was compared. Biomarkers identified as important by the algorithms were also analysed in relation to their involvement in the pathological processes.

**Results:**

Results showed that a high unbalance in the datasets significantly affected the classification by random forest and decision trees, leading to the overestimation of the sick class and low model performance. CACTUS algorithm was more robust to the unbalance in the dataset. CACTUS obtained a balanced accuracy of 0.747 for both genders, 0.718 for females and 0.803 for males. The analysis showed that in the classification process for the whole dataset: microalbumin, male gender, and tPSA emerged as the most informative biomarkers. For males: age, microalbumin, tPSA, cystatin C, BTA, HAD and S100A4 were the most significant biomarkers while for females microalbumin, IL-8, pERK, and CXCL16.

**Conclusions:**

CACTUS algorithm demonstrated improved performance compared with other methods such as decision trees and random forest. Additionally, we identified the most relevant biomarkers for the specific patient group, which could be considered in the future as novel biomarkers for diagnosis. Our results have the potential to inform future research and provide new personalised diagnostic approaches tailored directly to the needs of the individuals.

## 1 Introduction

Haematuria is defined as the visible presence of red blood cells (RBCs) in urine (gross haematuria) or at least three RBCs in high-powered field upon microscopic evaluation of a urine sample. Prevalence of microhaematuria among the general population is relatively high. It was estimated that in 2.4 -31.1% of total urine samples, RBCs are detectable in concentrations exceeding a fixed reference threshold (1–4). A haematuria patient population can be heterogenous with differences in age, gender, risk factors, geographical diversity etc., and it can have very different aetiology, including the presence of genitourinary malignant diseases. While most commonly cases of haematuria are non-malignant (e.g., infection, kidney or bladder stones, benign prostate enlargement, menstrual blood contamination), first-stage assessment should always be focused on the physical examination and collection of patient history, current treatment (e.g., anticoagulants) (5), lifestyle (e.g., smoking, alcohol consumption, strenuous physical activity), occupational hazards and risk factors (6). Dipstick urine analysis can be performed to confirm or exclude some causes of haematuria, for example, infection. For non-obvious cases, further investigation should be performed. Currently, cystoscopy together with urine cytology is the gold standard for bladder cancer diagnosis. Cystoscopy is an invasive procedure which is not without risk e.g., infection, bleeding, and pain. Computed tomography (CT) urography is warranted for patients who require upper urinary tract investigation, which raises concerns of radiation exposure (7). A retrospective study by Georgieva et al. (8) compared the benefits, harms, and costs of different haematuria evaluation guidelines and showed that guidelines which missed the fewest cancers also generated the highest number of radiation-induced cancers, false-positive cases, and diagnostic procedures costs (**Table 1**). They also showed that uniform CT imaging for patients is associated with a limited increase in cancer detection, high personal cost and is generally uneconomical.

**Table 1:** Comparison of different haematuria guideline outcomes simulated on the modelled haematuria patient’s cohort.

	Dutch Guidelines (9)		CUA Guideline (10)		KP Guidelines (11)		HRI Guidelines (6)		AUA Guidelines (4)	
Total urinary tract cancers	Cancer Detected	Cancer Missed	Cancer Detected	Cancer Missed	Cancer Detected	Cancer Missed	Cancer Detected	Cancer Missed	Cancer Detected	Cancer Missed
3514 (2980-4090)	3263 (2260-3240)	251 (140-400)	3343 (2300-3290)	172 (100-300)	3385 (2550-3600)	130 (60-270)	3399 (2740-3750)	116 (50-250)	3432 (2760-3850)	82 (0-80)
False-positive (CT, ultrasonography, or cystoscopy)	6452 (4040-9410)		6740 (4220-9820)		9099 (6270-12 450)		13 811 (10 800-17 170)		22 189 (17 520-27 370)	
Lifetime radiation-induced cancers	NA		NA		108 (34-201)		136 (62-229)		573 (184-1069)	
Costs (total US$)	44 254 (8112-129 435)		46 163 (8466-135 063)		51 920 (12 546-143 170)		59 751 (13 434-153 739)		93 886 (21 670-237 374)	

(Dutch Urological Association Guidelines, CUA, Canadian Urological Association; KP, Kaiser Permanente Program; HRI, Haematuria Risk Index; AUA, American Urological Association); NA, not applicable, based on (8).

Given the high prevalence of haematuria, the numerous potential causes, and the significant human and financial costs involved, the development of non-invasive diagnostic tests, based on biomarkers from urine or blood samples, would be a major step forward. However, this presents a significant challenge. To date, only two biomarkers - nuclear matrix protein (NMP22) and bladder tumour antigen (BTA) - have been approved by the Food and Drug Administration (FDA) for the detection and monitoring of bladder cancer. Unfortunately, commercially available tests for both biomarkers have low specificity and high false-positive rates (12, 13). Data shows that combining biomarker screening (NMP22) with cytology may improve patient screening (14), but current guidelines do not recommend the use of urinary tumour biomarkers or cytology in the initial evaluation of microhaematuria. To improve the diagnostic pathway, current research has focused on shifting towards a multi-biomarker approach. This approach has been proven to provide improvements in cancer detection (15, 16) while also being cost-effective in differentiating patients with benign and malignant disease (17). The complexity of the diverse causes of haematuria necessitates studies with a large number of possible biomarkers, with the associated challenge of identifying the most informative without creating false discoveries. This makes multi-biomarker studies more complex and less tractable, creating a need for computational tools to generate personalised insights from the available data (18–20).

Numerous studies have proved that by using advanced analytical methods, it is possible to create algorithms that can improve patient diagnosis with multiple biomarker analysis (15, 21, 22). Machine Learning (ML) (23, 24), especially, has been able to produce unique insights using different data sources (25–27). One of the major challenges of traditional ML models is poor generalisation, due in part to low robustness to unbalanced distribution of classes within a dataset, which is a common scenario in medical data. These models pay equal attention to the majority and minority classes. As a result, they often perform poorly on the minority class, especially when the imbalance in the data is extreme (28). Data dimensionality is another major challenge for ML algorithms, especially when dealing with small datasets where the number of features exceeds the number of samples and where different types of data (e.g. continuous or categorical) are present. Non-meaningful parameters need to be separated to subtract hidden information and provide actionable insights to clinicians. This could be achieved at the level of domain experts and data-driven features that could be incorporated into the model design. The final challenge for ML, and currently a requirement for any clinical decision support system, is explainability. Explainability is a property of an AI algorithm that allows a human to understand why a particular decision was made. In practice, explainability can either be an inherent property of an algorithm, or it can be approximated by other methods. Many modern ML methods can outperform humans in certain analytical tasks (e.g., pattern recognition), but they lack explainability, so the explanation must be approximated. On the other hand, the performance of traditional explainable methods is usually inferior to modern state-of-the-art methods such as neural networks, so the trade-off between performance and explainability is a major challenge for modern clinical decision support systems.

The Haematuria Biomarker (HaBio) dataset (22) is a unique collection of data illustrative of a patient population presenting with haematuria and includes an extensive range of biomarkers preselected based on literature searches and clinical experience. At the same time, HaBio presents all the challenges for ML described above. Considering the need for novel biomarker discovery for haematuria patients’ stratification and ensuring the models explainability, we analysed the HaBio cohort using various ML algorithms, including the recently developed CACTUS explainable classification algorithm (29, 30). To facilitate the diagnosis procedure and provide actionable insights for clinical patient management, we have provided a selection of biomarkers that could be useful in clinical practice, along with their possible decision boundaries.

## 2 Materials and methods

### 2.1 HaBio cohort

The HaBio Study was a three-way collaborative project between Queen’s University Belfast, Northern Ireland Health Trusts and Randox Laboratories Ltd.

**Figure 1:**
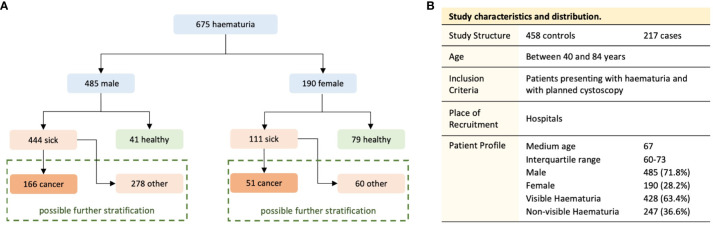
Division of the dataset into the study groups **(A)** and the structure of the study population **(B)**.

HaBio was funded by Invest Northern Ireland and Randox Laboratories Ltd. Ethical approval was obtained from the Office for Research Ethics Committee Northern Ireland (11/NI/0164) to recruit patients who satisfied the HaBio study inclusion criteria (22). The protocol for HaBio was also reviewed by hospital review boards and was conducted according to the Standards for Reporting of Diagnostic Accuracy (STARD) (31). A total of n=677 patients were recruited to HaBio, of which n=2 patients were excluded due to incomplete data. Therefore, the complete dataset is available for n=675 patients (n=485 males and n=190 females). There are significantly more males (2.5:1 ratio of males to females) which reflect “real world” urology patterns of presentation to haematuria clinics at the time of recruitment. This observation is borne out by the large number of men with benign prostatic hyperplasia (BPH) as a cause for haematuria. Within each gender there was a 2:1 ratio of non-cancer versus cancer (males 1.9:1 (319:166); females 2.7:1 (139:51), **Figure 1**).

### 2.2 Biomarker analysis

At the time of recruitment, a research nurse or clinician measured each patient’s height, weight and blood pressure while also recording details of medical history, lifestyle/behaviours, and occupations before collecting urine (25ml) and blood (35ml) samples. In the collected samples, 80 biomarkers previously indicated as potential biomarkers of urinary tract diseases, representing a range of biological pathways, were measured (**Supplementary Materials, Table A**). Patient samples were analysed in triplicate and the results were expressed as a mean ± SD.

In the study, chosen biomarkers were analysed with several different techniques. At recruitment, patient urine samples were collected prior to cytoscopic examination and evaluated using the POC test for NMP22 (BladderChek, Alere, US). Osmolarity (mOsm) was determined using a Löser Micro-osmometer according to manufacturer’s instructions (Löser Messtechnik, Berlin, Germany). Total urinary protein levels (mg/ml) were measured by Bradford assay (Pierce, Rockford, IL, USA). For multimarker analysis Biochip Array Technology was used (simultaneous detection of multiple analytes from a single patient urine and/or serum sample) (Randox Clinical Laboratory Services (RCLS), Antrim, Northern Ireland, UK), other biomarkers were measured using commercially available ELISA kits. Detailed description of analytical procedures is provided in **Supplementary Materials**. When data was below the Limit of Detection (LOD) or the Mean Detectable Dose (MDD) for any given test, 90% of the LOD or the MDD was used in lieu of the actual value for analysis (22).

### 2.3 Classical approach

In the study, due to differences in the typical causes of haematuria and the prevalence of malignant diseases we analysed data separately for male and female participants, in addition to the entire cohort. In the pre-processing step, as the data were characterised by a highly skewed distribution, we performed a log transformation of the biomarker measurement results for further analysis to reduce the skewness and replaced missing data with the median value for the given biomarker. For analysis, urine and serum biomarkers were used; if the same biomarker was analysed in both serum and urine samples, serum results are indicated by the word “serum” in the biomarker name.

**Figure 2:**
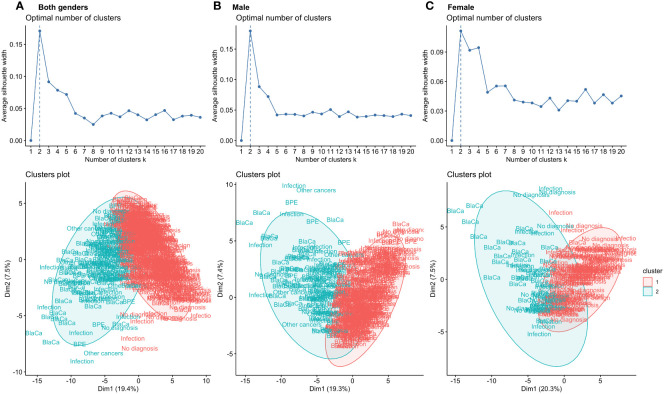
K-means algorithm applied to the input features for both genders **(A)**, males **(B)** and females **(C)** used for classification. For all data subsets, the highest silhouette score was obtained for k = 2. Visualisation of the clustering process showed overlap within the final diagnostic group of the clusters, indicating poor compactness in the clusters. Individual patients are presented as a description of the final diagnosis.

Firstly, we performed k-means clustering to assess if analysed features could be linearly separated. For k-means clustering, we iteratively tested the number of clusters from 1 to 20 and used the silhouette width to select the best configuration. We observed that for all three data subsets, the optimal value of clusters for k-means clustering was 2, showing that the distribution of features does not follow clear macro patterns or reflect the underlying number of causes of haematuria (**Figure 2**). As it was not possible to distinguish the number of clusters reflecting the number of underlying classes of final diagnosis, based on clinical evaluation and experience we decided to stratify patients into two subgroups, sick and healthy. The sick population had any of the following possible causes for their haematuria: chronic kidney diseases, infection, other benign diagnosis, bladder cancer, history of bladder cancer or other types of cancer (e.g., prostate cancer, renal cell carcinoma). The healthy population included every patient with no causes identified for their haematuria.

The initial analysis included logistic regression analysis and assessment of balanced accuracy for each biomarker separately. For logistic regression, we tested two approaches: linear and, to account for any possible non-linear relationship between the biomarkers and the outcome, we also fitted natural cubic splines. The results of the two approaches were later compared by ANOVA and the best performing model was selected as the final regression analysis. Afterwards, we applied binary decision trees and random forest models. For both models we performed 10-fold cross validation repeated three times. As the random forest is not an inherently explainable method, in contrast to the decision trees, we applied the local interpretable model-agnostic explanations (LIME) algorithm (32), to provide the explanation of the classification process and understanding of the biomarkers’ influence on the final class prediction. LIME focuses on explaining the model’s prediction for individual cases. LIME generates a new dataset consisting of perturbed samples and the corresponding predictions and then trains an interpretable model (regression) on this new dataset, weighted by the proximity of the sampled cases to the case of interest. Because a linear model is inherently interpretable, the fitted weights can be inspected and viewed as proxies for feature importance and based on the proximity of the values to the perturbed data point, the cut-off values for individual features can be provided.

All the analysis described in this section were performed in R (33).

### 2.4 CACTUS classification

To model the healthy and sick classes, we used the CACTUS (29, 30) algorithm. In the first step, fully anonymized data abstractions of the quantitative and qualitative biomarker data were generated by (34) transforming raw biomarker data into two-stage data abstractions (flips) based on receiver-operator curve (ROC) theory. These flips were encoded with the last letter of the label for each biomarker: up (U) abstracts raw data above, and down (D) below calculated cut-off values. For each biomarker, significance was determined from the node’s conditional probability *P*(ƒ|*c*_*i*_) of the flip ƒ, given the class *c*_*i*_ (sick or healthy). To assess how the conditional probability *P*(ƒ|*c*_*i*_), will change across the *N* considered classes, and to infer their importance for the classification process, ranks *(R_xf_)* were calculated for each biomarker according to the to the Equation 1.


(1) $$R_{x_{f}}=\;\frac{\sum_{i=1}^{N}\sum_{j>i}^{N}\left|P\left(x_{f}|c_{i}\right)-P\left(x_{f}|c_{j}\right)\right|}{N^{C_{2}}}$$

To assess the accuracy of the network’s patient classification we calculated the (Equation 2). For every patient in the given state “*s”* (sick or healthy) the cost function ( $C_{s})\;$ was calculated based on the corresponding node significance ( $\sigma_{s,i}$ ) of each biomarker ( $x_{i}$ ):


(2) $$C_{s}=\prod_{i}^{n}\sigma_{s,i}x_{i}$$

The cost function with the greater value was the determinant for patient classification as sick or healthy. Obtained classifications were compared to real diagnosis groups, marked as true positive (TP), false negative (FN), true negative (TN) or false negative (FN) and used to calculate specificity (Equation 3.a), sensitivity (Equation 3.b) and accuracy (Equation 3.c) for all tested models. Due to the much higher number of sick patients in our study groups, we used balanced accuracy (a metric which is robust for unbalanced datasets) to assess model performance.


(3a) $$Sensitivity=\frac{TP}{TP+FN}$$

(3b) $$Specificity=\;\frac{TN}{TN+FP}$$

(3c) $$Balanced\;accuracy=\frac{sensitivity+specificity}{2}$$

CACTUS has been implemented in Python3 (35).

### 2.5 Comparison of tested models

We evaluated the performance of each model using the χ2 test, which assesses whether the performance of the model is better than random chance. To compare the performance of the tested models, we performed a pairwise comparison of the model results using the McNemar test on the classification results, with a significance level of 0.05.

## 3 Results

K-means clustering was performed to assess how linearly separable the results were and whether it is possible to distinguish the final diagnostic group by the structure of the clusters. To select the best number of clusters, we used the silhouette score for which the highest value was obtained for two clusters in all analysed data subsets (*k = 2*). To visualise how patients with different final diagnosis groups are distributed within clusters, we have plotted individual points as biomarkers representing the final diagnosis on **Figure 2**. The graph shows that different final diagnosis groups are clustered within the same clusters, and a significant overlap of clusters.

Assessment of balanced accuracy of the logistic regression showed that single biomarkers were not specific enough to discriminate between healthy and sick patients (**Supplementary Materials, Tables B–D**). For both genders, the highest accuracy was obtained for urine cystatin C (0.580, cubic spline), soluble tumour necrosis factor receptor I (sTNFRI, 0.572, linear model), and progranulin (0.516, linear model). For females, the three biomarkers with the best scoring performance were phospho-extracellular signal regulated kinase (pERK, 0.673, linear model), microalbumin (0.672, linear model) and chemokine (C-X-C motif) ligand 16 (CXCL16, 0.667, linear model). In the male data subset, which is highly unbalanced, no single biomarker gave an accuracy higher than 0.5.

**Figure 3:**
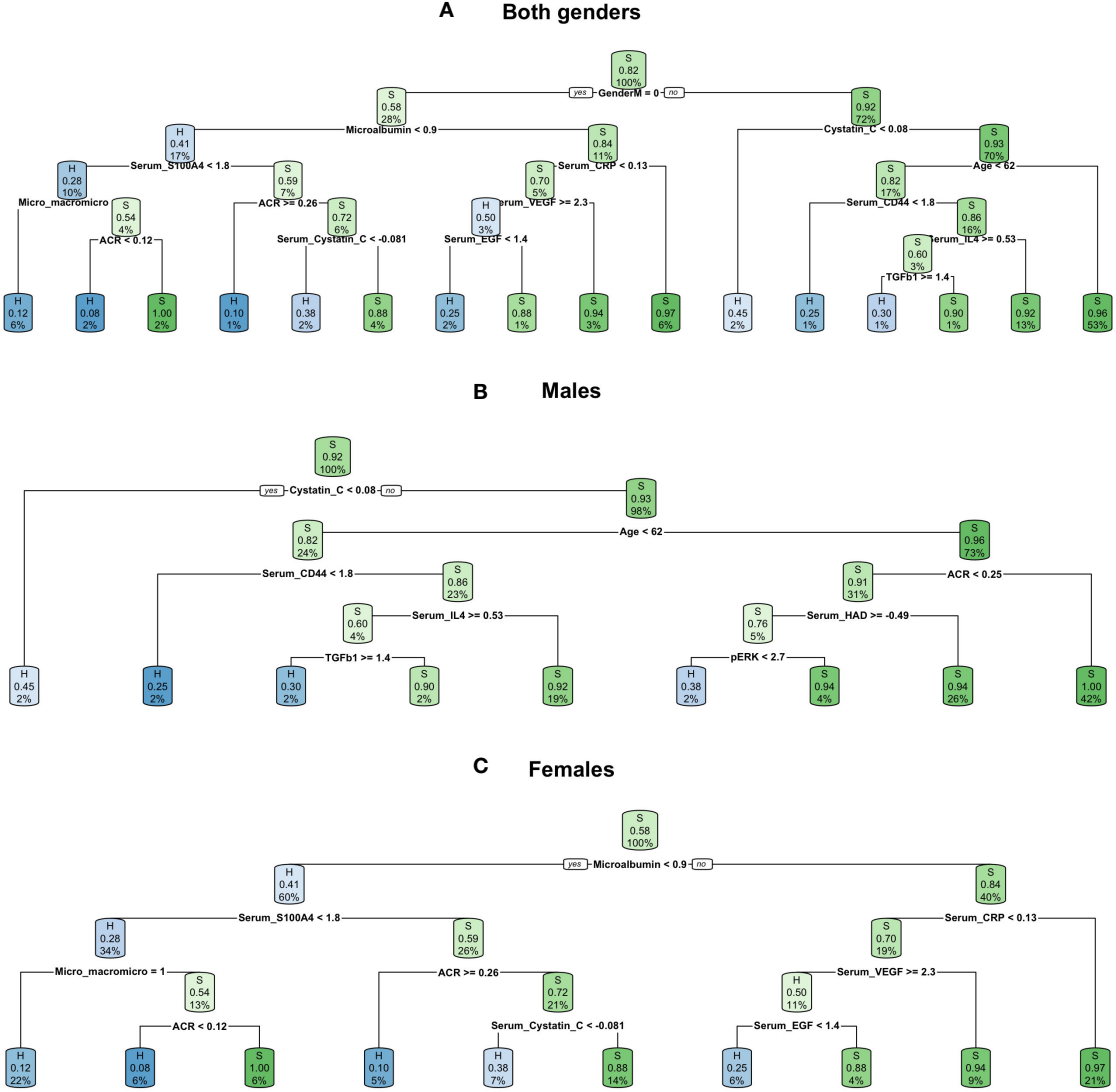
Decision trees created for all three datasets: **(A)** both genders dataset, **(B)** males dataset, **(C)** females dataset.

Decision trees provided simple rule-based models based on a maximum of 14 biomarkers (including gender) for patient classification. The most complicated tree was built for a dataset with patients of both genders. The first branch was built based on the male gender; resulting in the subsequent branches being gender specific. The male and female decision trees were similar to the branches of the tree built for both genders, with some additional branches. In the case of males, stratification was improved by adding decision boundaries based on serum hyaluronic acid (HAD) and pERK levels, allowing additional healthy individuals to be distinguished. In the female decision trees, the situation was reversed; the classification was performed with a lower number of features and some of the branches of the trees for both genders, such as vascular endothelial growth factor (VEGF), were pruned. The highest balanced accuracy of decision tree classification was obtained when both genders were analysed together (0.640, **Figure 3A**), even though the first split was on gender. Separate stratification for males and females gave lower balanced accuracy (0.551, **Figure 3B** and 0.623, **Figure 3C**), and better significance and specificity was obtained for females as the data subset was more balanced (**Table 2**).

**Table 2:** Comparison of tested models’ performance, statistical analysis was performed with the χ2 test, with significance level of 0.05.

CACTUS					
	Accuracy	Balanced Accuracy	Sensitivity	Specificity	p-value
**Both**	0.751	0.747	0.753	0.742	< 2.2e-16
**Male**	0.781	0.803	0.777	0.829	1.944e-11
**Female**	0.716	0.718	0.703	0.734	3.232e-09
Decision trees					
	Accuracy	Balanced Accuracy	Sensitivity	Specificity	
**Both**	0.822	0.640	0.922	0.358	< 2.2e-16
**Male**	0.894	0.551	0.963	0.140	3.238e-4
**Female**	0.632	0.623	0.676	0.570	1.898e-09
Random Forest					
	Accuracy	Balanced Accuracy	Sensitivity	Specificity	
**Both**	0.853	0.627	0.978	0.275	< 2.2e-16
**Male**	0.918	0.512	1.000	0.024	0.135
**Female**	0.690	0.665	0.812	0.519	3.808e-06

**Figure 4:**
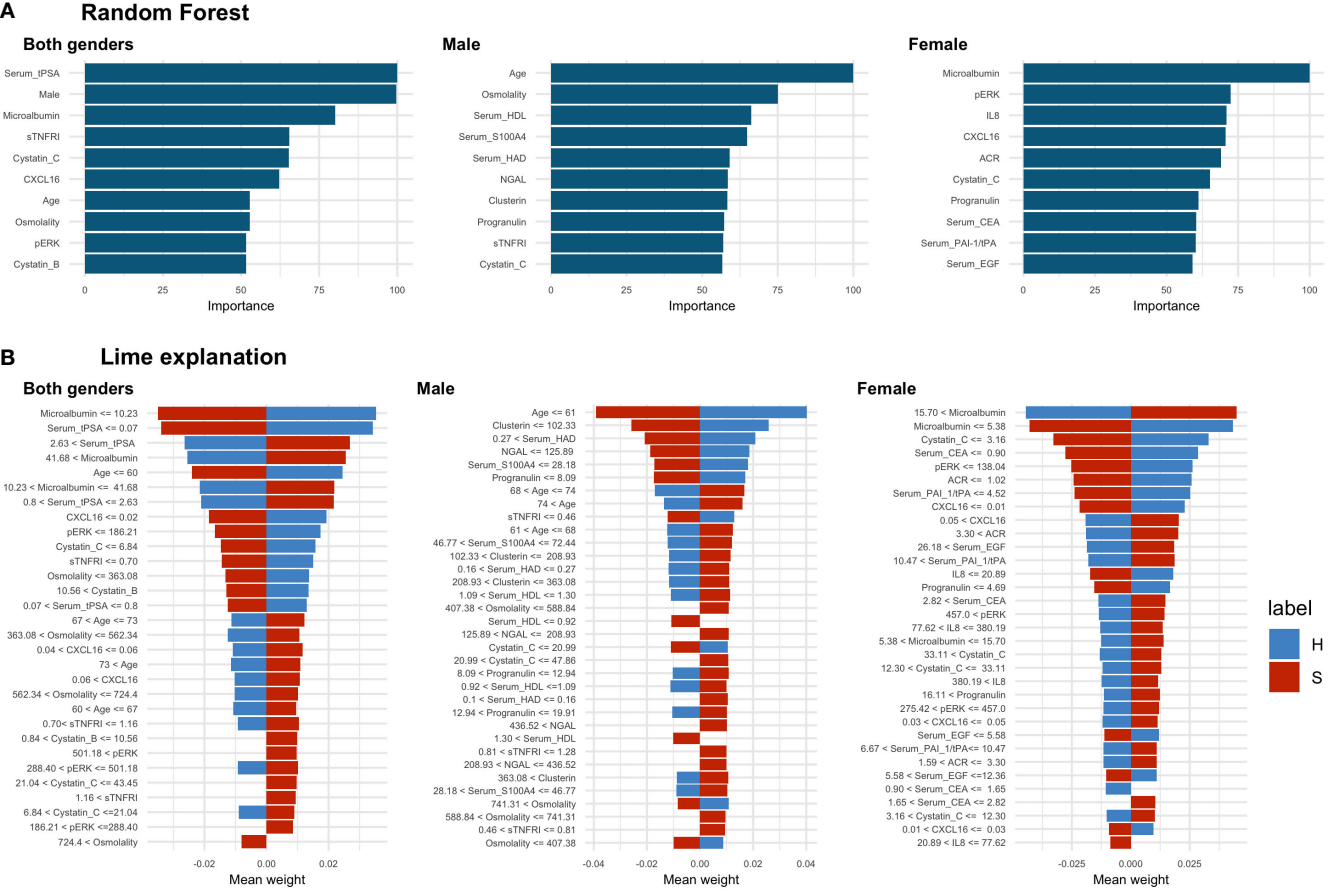
Random forest results. **(A)** Top 10 most important features selected by random forest, **(B)** LIME analysis results, the cut-off values are presented as raw values for each biomarker. The value of the mean weight of the biomarkers indicates whether the biomarker, within the specified range, has a positive (positive value) or negative (negative value) influence for being classified as a sick (S) (red colour) or healthy (H) (blue colour) individual. In the case of both genders’ dataset analysis there were 4 biomarkers with a positive and 1 with a negative influence on sick class. In the male data subset, there were 9 biomarkers with a positive and 2 biomarkers with a negative influence on the sick class. In the female data subset, there was 1 biomarker with positive and 1 with negative influence towards the sick class and 1 with positive influence towards the healthy class.

The effectiveness of the random forest classification was also insufficient to discriminate between sick and healthy individuals (**Table 2**). The highest value of balanced accuracy was obtained for the female data subset (0.665), lower for both genders (0.627) and the lowest for the male subset (0.512, not statistically significant, p-value = 0.135). The corresponding values of sensitivity and specificity showed a bias towards the more prevalent class (sick), which is most visible in the case of the male data subset (sensitivity: 1.00, specificity: 0.024), showing that all cases of sick individuals were correctly classified and only one healthy individual was correctly classified. We also extracted the top 10 features for random forest classification (**Figure 4A**). As can be seen in the graph, two of the most important features are gender-specific (serum total prostate specific antigen (tPSA) and gender), justifying the need to separate female and male cases for analysis purposes. Several biomarkers such as: microalbumin, osmolarity, sTNFRI, cystatin C, CXCL16, pERK, progranulin, and patient age were of high importance for two or three data subsets. Although the biomarkers were common to the data subsets, as the LIME analysis showed, the decision boundaries (levels of the biomarkers) and their contribution (weights) to the final model were different. For example, for age, which is one of the most important characteristics, the lowest cut-off value in the case of both genders was 60 and has a slightly higher influence on the classification of the healthy category than the sick category; for the male data subset only, this cut-off value was 61, with the same influence on the classification. In the case of all analysed data subsets, there were some biomarkers within certain ranges that had a clear positive or negative influence on the classification (**Figure 4B**, and **Table 3**).

**Table 3:** Comparison of the decision boundaries for each algorithm. The cut-off values are shown as raw measurement results.

Marker	CACTUS			Random Forest (LIME)			Decision Tree		
	Both	Male	Female	Both	Male	Female	Both	Male	Female
**ACR**	–	1.74	–	–	–	(1.02,1.59] (1.59,3.30]	1.31 1.82	1.77	1.31 1.82
**Age**	–	63	–	(60,67] (68,73]	(61,68] (68,73]	–	61.5	61.5	–
**BTA**	–	9.92	–	–	–	–	–	–	–
**Clusterin**	–	–	–	–	(102.33, 208.93] (208.93, 363.08]	–	–	–	–
**Creatinine**	–	–	48.39	–	–	–	–	–	–
**Cystatin B**	–	–	–	(0.84, 10.56]	–	–	–	–	–
**Cystatin C**	–	–	–	(6.84,21.04] (21.04, 43.45]	(20.99, 47.86]	(3.16, 12.30] (12.30, 33.11]	1.20	1.20	0.83
**CXCL16**	0.03	–	0.02	(0.02, 0.04] (0.04, 0.06]	–	(0.01, 0.03] (0.03, 0.05]	–	–	–
**D-dimer**	–	16.03	–	–	–	–	–	–	–
**Gender**	M	–	–	F, M	–	–	M	–	–
**HAD**	–	–	–	–	(0.10, 0.16] (0.16, 0.27]	–	–	0.32	–
**Haematuria**	–	–	–	–	–	–	micro	–	micro
**IL1a**	–	–	2.26	–	–	–	–	–	–
**IL7**	1.59	–	–	–	–	–	–	–	–
**IL8**	–	–	68.12	–	–	(20.89, 77.62] (77.62, 380.19]	–	–	–
**MCP_1**	46.35	47.33	–	–	–	–	–	–	–
**Microalbumin**	7.87	13.43	7.87	(10.23, 41.69]	–	(5.38, 15.70]	7.90	–	7.90
**Midkine**	144.76	–	68.12	–	–	–	–	–	–
**NGAL**	–	–	–	–	(125.89, 208.92] (208.92, 436.52]	–	–	–	–
**pERK**	–	–	224.38	(186.21, 288.40] (288.40, 501.19]	–	138.04, 275.42] (275.42, 457.0]	–	475.75	–
**Osmolarity**	–	–	–	(363.08, 562.34] (562.34, 724.44]	(407.38, 588.84] (588.84, 741.31]	–	–	–	–
**Progranulin**	6.72	–	6.72	–	(8.09, 12.94] (12.94, 19.91]	(4.69, 16.11]	–	–	–
**serum_CD44**	–	–	–	–	–	–	65.22	65.22	–
**serum_CEA**	–	–	–	–	–	(0.90, 1.65] (1.65, 2.82]	–	–	–
**serum_CRP**	–			–	–	–	1.33	–	1.33
**serum_Cystatin C**	–	0.98	–	–	–	–	0.84	–	–
**serum_EGF**	–	–	–	–	–	(5.58, 12.36] (12.36, 30.20]	23.05	–	23.05
**serum_HDL**	–	–	–	–	(0.92, 1.09] (1.09, 1.30]	–	–	–	–
**serum_IL4**	–	–	–	–	–	–	3.36	3.36	–
**serum_PAI_1/tPA**	–	–	6.23	–	–	(4.52, 6.67] (6.67, 10.47]	–	–	–
**serum_S100A4**	–	–	–	–	(28.18, 46.77] (46.77, 72.44]	–	63.75	–	63.75
**serum_tPSA**	0.37	1.03	–	(0.07, 0.80] (0.80, 2.63]	–	–	–	–	–
**serum_VEGF**	–	–	–	–	–	–	221.68	–	221.68
**sTNFRI**	0.3	–	0.29	(0.7, 1.16]	(0.46, 0.81] (0.81, 1.28]	–	–	–	–
**sTNFRII**	–	0.34	–	–	–	–	–	–	–
**TGFβ1**	–	–	–	–	–	–	26.78	26.78	–
**Urinary Protein**	0.104	0.103	–	–	–	–	–	–	–

For LIME results, only the mid-ranges are shown (upper and lower ranges are presented in **Supplementary Materials, Table E**). The decision boundaries made by LIME go as follows: below the indicated range, as a range is presented in the table, and above the indicated range. The unit of the values presented goes as follows: BTA (U/ml), serum_CD44 (ng/ml), serum_CEA (ng/ml), Clusterin (ng/ml), Creatinine (mmolL), serum_CRP (mg/ml), CXCL16 (ng/ml), Cystatin B (ng/ml), Cystatin C (ng/ml), serum_Cystatin C (ng/ml), D-dimer (ng/ml), EGF (pg/ml), serum_HAD (U/l), serum_IL-4 (pg/ml), IL-7 (pg/ml), IL-8 (pg/ml), MCP-1 (pg/ml, Microalbumin (mg/l), Midkine (pg/ml), NGAL (ng/ml), Osmolarity (mOsm), pERK (pg/ml), Progranulin (ng/ml), serum_tPSA (ng/ml), Protein (mg/ml), serum_S100A4 (ng/ml), TGF-b1 (pg/ml), sTNFRI (ng/ml), serum_VEGF (pg/ml), serum_HDL (mmol/l). "-" biomarker was not selected by given algorithm.

CACTUS classification gave a higher balanced accuracy than the models described above for all analysed data subsets (0.747 both genders, 0.803 males, 0.718 females). Moreover, the obtained values of sensitivity and specificity were more balanced, although the sensitivity was lower, which indicated a higher false negative rate. The CACTUS specificity was higher than the specificity for decision trees and random forests, showing that the classification was not biased towards the predominant group (sick individuals) (**Table 2**).

**Figure 5:**
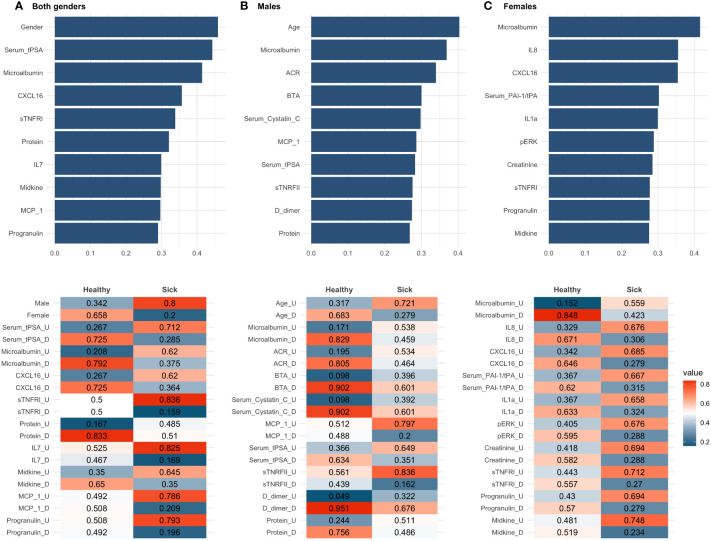
CACTUS analysis results for all three data subsets: **(A)** both genders dataset, **(B)** males dataset, **(C)** females dataset. The top panel presents the ten most important biomarkers according to the rank values. The bottom panel presents probability of flips for sick and healthy in descending order of rank value.

The 10 biomarkers with the highest CACTUS ranks for sick and healthy individuals in all groups is shown in **Figure 5**. The ranks provide information about the average difference between the classes (sick and healthy) for the probability of the biomarkers being in each state (‘U’ or ‘D’), meaning that the higher the rank value, the greater the difference in at least one of the probabilities. Like random forest, CACTUS confirmed the need to stratify patients into subgroups based on gender, as gender was indicated by CACTUS as the most important factor for the whole population studied. Additionally, the second most important biomarker was serum tPSA, a gender-specific biomarker of prostate health and therefore important in the classification process. Microalbumin was reported as the third most important biomarker for both genders, but also received a high score in the gender stratified analysis (second for men and first for women).

In the male population, age is the highest ranked factor and was not reported in any of the other subsets analysed. In addition, as in the random forest analysis, several biomarkers such as serum tPSA, microalbumin, CXCL16, urinary protein, monocyte chemoattractant protein-1 (MCP-1), and progranulin were present with a high score in more than two groups and are therefore more sensitive to discovering the differences in flips which were more prevalent in the healthy class, than the sick class. This was visible as a higher difference in between probabilities of nodes in the healthy. Interestingly, microalbumin was the only common biomarker in both male and female results, suggesting a gender-specific mechanism for haematuria development.

In CACTUS analysis, the flip probabilities indicated whether the biomarker was generally below (‘D’) or above (‘U’) the calculated cut-off values; we observed that the distribution of some of the biomarkers changed significantly between classes. For example, when looking at the dataset for both genders, we can see that male, having a serum tPSA above the cut-off value and having high levels of microalbumin are important factors for classification as sick. We have also observed that some features were only important for classification in one class and for the second class there was an equal or almost equal probability of the flip probabilities. This was the case in the both genders dataset for sTNFRI, which had the same probability of flip for healthy individuals (0.5, 0.5), but showed higher probability (0.836) for sTNFRI being in state (‘U’) for the patients classified as sick. In the male population, the probability of flips indicated that being older (0.721), with higher levels of MCP-1 (0.797), serum tPSA (0.649) and sTNFRII (0.836) and reduced levels of D-dimer (0.676) were important factors in being sick. It is interesting to note that in the male population, CACTUS classification detected higher differences in the flip’s probability for healthy individuals than in sick. In the female dataset we observed the gradual change in the flip probabilities, i.e., the highest difference in the flip probability, which was most important for healthy individuals (microalbumin), had at the same time the lowest difference for sick individuals and vice versa.

## 4 Discussion

In the study, we analysed the HaBio cohort, which contains data from patients presenting with haematuria. One of the challenges related to the analysis of this dataset is the unbalanced structure of data, both in terms of gender (male predominance) and the different number of patients in each disease category. The data structure reflects the real-world structure of the patients reporting to a clinician with haematuria and is related to differences in diagnostic processes and potential risks. Males, older patients, and smokers have significantly higher malignancy risk (36–39). On the other hand, women do not receive the same diagnostic attention, which leads to delays in urological consultation and poorer oncological outcomes in bladder cancer (22, 40, 41). It is therefore crucial to provide gender-specific blood or urine biomarkers which could reduce the time and harm associated with the current methods, while being affordable and addressing gender inequalities in the diagnostic process.

A second element contributing to the unbalanced structure of the dataset was the different number of patients in each category. As k-means clustering showed it was not possible to distinguish the final diagnostic group (bladder cancer, benign prostate enlargement, infections, incidental haematuria, other cancers, and benign disease) by the structure of the clusters. The best results were obtained when clustering into two, highly unbalanced clusters (**Figure 2**). Although, it is possible to computationally balance datasets during analysis (42–44) these data reflect the true distribution of patients presenting to clinicians with haematuria, so no pre-processing techniques were used to balance the class distribution. Additionally, initial stratification into healthy and sick groups could expedite the diagnostic process by referring patients to the most appropriate specialist or for more targeted diagnostic and less invasive testing, which could be beneficial for patients and clinicians.

In the case of decision trees and random forests we observed a strong influence of the unbalanced nature of the dataset on the classification process, while CACTUS was the most robust. As the imbalance between sick and healthy increased (111:79 for females, 152:523 for both genders and 41:444 for males) the discrepancies between the model metrics (specificity, sensitivity, accuracy, and balanced accuracy) also increased (**Table 2**). This was particularly evident for the male random forest analysis, where the balanced accuracy was 0.512 and the specificity 0.024, meaning that in this case only one healthy individual was classified as healthy. This result was not statistically significant, meaning that there was no difference between the classification result and random chance. A possible explanation for this was that random forests build each constituent tree from a bootstrap sample of the training data. There was a significant chance that bootstrapped samples from extremely unbalanced datasets could contain few or even none of the minority class, resulting in a model with poor performance. On the contrary, CACTUS despite the high prevalence of sick classes in the males dataset, obtained very high specificity (0.829), meaning that the algorithm was able to detect a high number of healthy patients and could potentially exclude them from subsequent invasive diagnostic procedures. The high performance of CACTUS was a result of its design. The classification process was based on the probability of each feature being in the state “U” or “D” for the given class which was not influenced by the number of cases in each class. Therefore, when the imbalance was high (both genders and males dataset) CACTUS generates the statistically significant improvement in the classification (**Table 4**) when comparing to random forest and decision trees.

**Table 4:** Pairwise comparison of the model’s performance for the data subsets with McNemar test.

Both genders	Decision trees	Random forest
**Cactus**	2.2e-16	2.2e-16
**Decision trees**		2.51e-05
Males	Decision trees	Random forest
**Cactus**	< 2.2e-16	< 2.2e-16
**Decision trees**		0.088
Females	Decision trees	Random forest
**Cactus**	0.088	4.93e-07
**Decision trees**		0.078

Logistic regression, showed that single biomarkers were not effective in identifying sick or healthy patients (**Supplementary Materials, Tables B-D**). It has been shown that the use of multiple biomarkers can improve the stratification of patients with bladder cancer (22), which has been confirmed by our analysis. The highest improvement in balanced accuracy was obtained for the male subset with the CACTUS classifier (0.803 versus 0.500 for single biomarker analysis). We also observed improvements in balanced accuracy for both genders (from 0.572 for the best single marker to 0.747) and for females (from 0.672 to 0.718) when using CACTUS. Interestingly, this improvement was not as significant using the other two methods (**Table 2**).

The aim of the study was not only to classify patients, but also to identify potential biomarkers and their decision boundaries (**Table 3**). As shown in **Table 3**, the most important biomarkers differ widely between the algorithms and the data subsets tested. For the dataset of both genders, the most important features for all algorithms were gender and microalbumin. Microalbumin has been described in the literature as a marker of renal dysfunction (45, 46). There is some evidence that elevated levels of microalbumin may be associated with some types of cancer, including cancer of the urinary tract (47). In the literature, values of microalbumin below 20 mg/mL are considered physiologically normal, but according to our analysis, the decision boundaries could be much lower, >5.38 mg/mL or >13.43 mg/mL depending on the model and dataset (**Table 3**). The values above the decision boundaries are classified as important for the stratification process and are more indicative of sick individuals. Therefore, when using the official reference values, it is possible to miss some individuals with developing pathology.

Another important biomarker selected by random forest and CACTUS algorithms for both genders dataset was serum tPSA. The decision boundaries for serum tPSA were underestimated when analysed in the both genders dataset due to the presence of female samples, where in most cases the serum tPSA level was below the detection limit. For the male dataset, serum tPSA was only indicated by CACTUS, with a level of 1.03 ng/mL being indicative of a pathological state. This is well below the reference values even in the youngest men. PSA is prostate specific antigen and elevated levels of PSA could be caused by conditions that lead to disruption of the epithelial cells of the prostate basal membrane, such as prostatitis, benign prostatic enlargement (BPE), prostate biopsies and surgery or decreased by medication, including 5-alpha reductase inhibitors (48–51). As the male dataset includes patients with different underlying causes of haematuria, not all of which affect PSA levels, observed values may be lower than reference levels even in the presence of BPE in the study group. Gender stratification is also strongly associated with age, which was identified as one of the important features by decision trees when analysing the whole dataset, and by CACTUS and random forest when analysing males only. It is known that biomarkers (such as cytokines, lipids or organ-specific biomarkers such as PSA (52–54)) change with age, as does the likelihood of developing age-related conditions such as prostate enlargement (55) or bladder cancer (56). According to our results patients over 60 years of age, and especially males over 63 (CACTUS estimation) should receive special attention during the diagnostic process, as risk of developing disease increases. These results are in line with current American Urological Association (AUA) guidelines (4), which place male patients over the age of 60 at high risk of malignancy.

Several biomarkers important for bladder cancer screening were also indicated in the models in the male dataset, i.e., BTA (CACTUS), HAD (random forest and decision tree), and S100 calcium-binding protein A4 (S100A4) (random forest), however they were not among the most important features in the respective models. This may be due to the wide variety of diseases underlying haematuria in the datasets. BTA (12, 13), HAD (57) and S100A4 (58) are closely related to tumourigenesis, so the addition of samples from individuals without malignant disease could influence the distribution of these features, making them less important for the classification process.

For the female stratification process, many of the most important characteristics differ from the male and both genders datasets. One of the selected biomarkers is interleukin-8 (IL-8) measured in urine. IL-8 is an angiogenic factor associated with inflammation and carcinogenesis. It has been shown that elevated urinary levels of IL-8 are associated with urothelial cell carcinoma (59, 60). In the study of Urquidi et al. (61) it has been shown that the urinary level of IL-8 in patients is elevated when compared to healthy controls with the median value of 128.43 pg/ml vs. 0 pg/ml, respectively. Our analysis set the decision boundaries at 68.12 pg/ml (CACTUS) and above 20.89 pg/ml (random forest), which is comparable to previously obtained data and allows a more detailed classification of patients. It is important to note that IL-8, as a pro-inflammatory cytokine, is also elevated in the samples of patients with urinary tract infections (59), so it should be used more as a biomarker of pathological conditions rather than specific diseases.

Other biomarkers that are important in stratifying women are the phosphorylated form of ERK and epidermal growth factor (EGF). ERKs are members of the mitogen-activated protein kinase (MAPK) family and are involved in cell cycle regulation and tissue proliferation. MAPK signalling is active in both early and advanced stages of tumourigenesis and promotes tumour proliferation, survival, and metastasis (62). EGF has also been shown to activate the MAPK/ERK pathway (63, 64). EGF, acting through the EGF receptor, promotes cancer development (65). EGF has been shown to promote bladder cancer cell proliferation (66). To the best of our knowledge, this is the first time that EGF has been described as a potential biomarker for the detection of the pathology related to urinary tract cancer, providing an initial estimate of the possible concentration of the biomarker for decision making.

Several biomarkers were common to more than one group including CXCL16, cystatin C and microalbumin (described above). CXCL16 is a cholesterol receptor and a chemokine with a potential role in vascular injury, angiogenesis, and inflammation. CXCL16 has previously been described to be elevated in patients with urothelial cancer (67, 68) and diabetic kidney disease (69). As CXCL16 is not a routinely studied biomarker, reference values for it have not yet been described, but according to our studies, elevated levels are associated with the pathological causes of underlying haematuria. Urinary levels of CXCL16 higher than 0.1 ng/mL or 0.3 ng/mL (depending on the gender and the model, **Table 3**), may be of use in the stratification of patients presenting with haematuria.

Cystatin C was also suggested as a potential biomarker by several models, when measured in urine and serum (**Table 3**). Cystatin C is a biomarker produced by all nucleated cells and is freely filtered by the kidney with almost complete reabsorption in the proximal tubule and no significant urinary excretion. It has been postulated that serum cystatin C levels may be a more stable alternative to creatinine for glomerular filtration rate (GFR) (70) and a potential new biomarker of renal dysfunction (71, 72). In addition, some studies have shown that decreased serum cystatin C levels may be present in bladder cancer (73). There is also some evidence of increased expression of *CST3* mRNA in higher-risk prostate cancer patients compared with those at lower-risk (74), but the utility of cystatin C (both serum and urine) requires further study. Our analysis showed that upper decision boundary for urinary cystatin C levels could be set up between 0.83 ng/ml and1.2 ng/ml (decision trees) and 6.84 ng/ml (random forest) are indicative of disease status. For men, the values are 1.2 ng/ml and 20.99 ng/ml (depending on the gender and the model, **Table 3**). As there are no officially established values for urinary cystatin C, reference values have been suggested at the level of 0.119-0.213 mg/L (75) or 0.06 - 0.16 mg/L (76) which is much higher than our study suggested. There are well established reference values for serum cystatin C which are around 0.58 - 1.02 mg/L (77). This is similar to the decision limits given by CACTUS for males (0.98 mg/L) and decision trees for both genders (0.84 mg/L).

Other biomarkers which were not common to all datasets or were only indicated by one of the algorithms are involved in different biological pathways, including inflammation (C-reactive protein (CRP), HAD, IL-8, MCP-1, Midkine, sTNFRI, neutrophil gelatinase-associated lipocalin (NGAL)) and metastasis (cluster of differentiation 44 (CD44), carcinoembryonic antigen (CEA), cystatin B, IL-8, NGAL, transforming growth factor beta-1 (TGF-β1)) which, after additional investigation, could also lead to the discovery of new clinically useful biomarkers.

In the study, we identify several biomarkers that have not been studied in relation to haematuria, or biomarkers without established reference values. Although this is a retrospective study, it may point the way for future research. We believe that several of the selected biomarkers (CXCL16 for both genders, HAD and S100A4 for males and IL-18, pERK and EGF for females) may have the potential to be introduced into routine diagnostics in the future, but this will require further work not only to establish reference values but also to better understand underlying mechanisms.

Notably some guidelines no longer recommend invasive testing for microscopic haematuria, and this seems to improve general patient management (78, 79). Given the challenges described in the diagnostic process, including the high cost (economic and personal) the proposed pre-stratification of patients with biomarker screening could be a further improvement. However, for people with macroscopic haematuria, cystoscopy is still recommended. In the HaBio cohort, 48% of patients with macroscopic haematuria did not have malignancy and had to undergo invasive diagnosis. Non-invasive methods based on biomarker screening could change the approach to the initial assessment of haematuria, reducing the number of false-positive and false-negative cases and providing affordable and time-efficient diagnostic procedures.

## 5 Conclusions

In this work, we addressed the challenging problem of diagnosing patients presenting with haematuria into two subclasses (healthy or sick), which could enable the introduction of improvements in patient management, allowing for a more efficient use of healthcare resources. With multiple possible causes and large variations in the number of patients with each condition, we addressed the problem of analysing unbalanced datasets in a medical setting and showed that by carefully selecting the models applied, it is possible to perform meaningful analysis even on challenging datasets. We focused on both classification and explanatory power to aid decision making. Although we were able to classify patients with satisfactory accuracy and provide decision boundaries for each of the biomarkers, our analyses were based on a retrospective study and further work is required to introduce the proposed biomarkers into clinical practice. Nevertheless, the classification obtained and the selection of biomarkers provided could be used to inform guidance for healthcare professionals to develop less invasive, faster and more economical strategies for patient disease management.

## Data availability statement

The datasets generated during and/or analyzed during the current study are not publicly available for privacy reasons, but are available on reasonable request. Requests to access these datasets should be directed to Mark W. Ruddock, mark.ruddock@randox.com.

## Ethics statement

The studies involving humans were approved by Office of Research Ethics Committee Northern Ireland (11/NI/0164). The studies were conducted in accordance with the local legislation and institutional requirements. The participants provided their written informed consent to participate in this study.

## Author contributions

AD: Conceptualization, Formal analysis, Methodology, Supervision, Validation, Visualization, Writing – review & editing. BD: Conceptualization, Investigation, Writing – review & editing. MR: Conceptualization, Investigation, Writing – review & editing. CR: Conceptualization, Investigation, Writing – review & editing. MK: Conceptualization, Writing – review & editing, Investigation. JW: Conceptualization, Investigation, Writing – review & editing. AI: Writing – review & editing. JL: Conceptualization, Investigation, Writing – review & editing. PF: Conceptualization, Investigation, Writing – review & editing. DO: Conceptualization, Investigation, Writing – review & editing. DC: Writing – review & editing, Investigation, Conceptualization. ME: Writing – review & editing, Conceptualization, Investigation. RB: Conceptualization, Investigation, Writing – review & editing. JS: Conceptualization, Formal analysis, Methodology, Software, Supervision, Writing – original draft.
