# Supplementary material for: Stratifying risk of disease in haematuria patients using machine learning techniques to improve diagnostics
Source: Front Oncol. 2024 May 8;14:1401071. doi: 10.3389/fonc.2024.1401071 (PMC11109371; doi:10.3389/fonc.2024.1401071)
Supplement: Supplementary file 1 [file DataSheet_1.docx]

**Supplementary Materials**

1. **Data collection**

**The Haematuria Biomarker Study Description**

The Haematuria Biomarker (HaBio) Study is a three-way collaborative project between Queen's University Belfast, Northern Ireland Health Trusts and Randox Laboratories Ltd. HaBio was funded by Invest Northern Ireland (INI) and Randox Laboratories Ltd. Ethical approval was obtained from the Office of Research Ethics Committee Northern Ireland (11/NI/0164) to recruit 677 patients who satisfied the HaBio study inclusion criteria.

**Inclusion and Exclusion Criteria**

**Bladder cancer patients:**

- Written informed consent to participate in the study
- Aged between 40 and 80 years
- Current haematuria or a history of haematuria
- Cystoscopy within the last 6 months or planned cystoscopy
- No chemo- or radio- therapy in the three weeks prior to recruitment
- No previous history of cancers other than bladder cancer
- Suspicion of bladder cancer or proven bladder cancer

**Control patients:**

- Written informed consent to participate in the study
- No previous history of cancer
- Of the same gender, approximate age, and smoking status (where possible) to a bladder cancer patient already recruited to HABIO
- Current haematuria or a history of haematuria
- Negative cystoscopy within the last 3 months, but at least 48h after the procedure
- No chemo- or radio- therapy in the three weeks prior to recruitment

**Exclusion Criteria**

**Bladder cancer patients:**

- No written informed consent to participate in the study
- Aged < 40 or > 85 years
- No history of haematuria
- No recent or planned cystoscopy
- Chemo- or radio- therapy in the three weeks prior to recruitment
- Previous history of cancer(s), other than bladder cancer
- No suspicion of bladder cancer or proven bladder cancer

**Control patients:**

- No written informed consent to participate in the study
- Previous history of any cancer
- Not of the same gender, approximate age and smoking status of a patients already recruited as a bladder cancer patient
- No history of haematuria
- No recent or planned cystoscopy
- Chemo- or radio- therapy in the three weeks prior to recruitment

The protocol for HaBio was also reviewed by hospital review boards and was conducted according to the Standards for Reporting of Diagnostic Accuracy (STARD) (Bossuyt, 2003). At recruitment, patient urine samples were collected prior to cryptoscopic examination and evaluated using the point of care test (POCT) NMP22 (BladderChek, Alere), according to manufacturer’s instructions (MDD <10 U/ml are negative). Additionally, at the time of recruitment, a Research Nurse (RN) or clinician measured each patient’s height, weight and blood pressure and recorded details about medical history, lifestyle/behaviours and occupations before collecting urine (25ml) and blood samples (35ml) for biomarker assessment. Biomarkers were assessed across a range of pathways **(Table 1).**

1. **List of analysed biomarkers**

**Table 1. Detailed list of the 80 biomarkers and their pathways in HaBio**

8-OHdG - 8-Hydroxydeoxyguanosine, ACR – Albumin:Creatinine Ratio, BTA - Bladder Tumour Antigen, CD44 – Cell Surface Glycoprotein, CEA - Carcinoembryonic antigen, CK-18 - Cytokeratin 18, CK-20 – Cytokeratin-20, CRP -  C-Reactive Protein, CXCL16 - Chemokine (C-X-C motif) Ligand 16, EGF - Epidermal Growth Factor, FABP-A - Fatty-Acid Binding Protein-Adipocyte, FAS - Fas Cell Surface Death Receptor, GRO-α - Chemokine Ligand 1, HDL – High Density Lipoprotein, HAD - Hyaluronidase, IFNɣ – Interferon Gamma, IL - Interleukins, IL-12p70 – Interleukin-12p70, LDL – Low Density Lipoprotein, LASP1 - LIM and SH3 Domain Protein 1, M2PK – Muscle Type-2 Pyruvate Kinase, M30 – M30 Apoptosense, MCP-1 - Monocyte Chemoattractant Protein-1,  MMP-9 - Matrix Metalloproteinase-9, MMP-9/NGAL complex – Matrix Metalloproteinase-9/Neutrophil-Associated Gelatinase Lipocalin Complex, MMP-9/TIMP-1 – Matrix Metalloproteinase-9/Tissue Inhibitor of Metalloproteinases-1 Complex, NGAL - Neutrophil-Associated Gelatinase Lipocalin, NMP22  - Nuclear Matrix Protein 22, NSE - Neuron Specific Enolase, pERK - Phospho-Extracellular Signal Regulated Kinase, PAI-1/tPA - Plasminogen Activator Inhibitor Type-1/Tissue Plasminogen Activator Complex, PSA/tPSA – Prostate-Specific Antigen/total Prostate-Specific Antigen, TGF-β1 - Transforming Growth Factor-β1,  S100A4 - S100 Calcium Binding Protein A4, sIL-2Ra – Soluble Interleukin-2 Receptor Alpha Chain (CD25), sIL-6R – Soluble Interleukin-6 Receptor (CD126), TNF-α - Tumour Necrosis Factor-Alpha, sTNFRI and II - Soluble Tumour Necrosis Factor Receptor-1 and -2, TM - Thrombomodulin, tPA - Tissue Type Plasminogen Activator, VEGF - Vascular Endothelial Growth Factor.

**Table A. Biological pathways associated with measured biomarkers.**

| **Pathways** | **Biomarkers** |
| --- | --- |
| Apoptosis | CK-18, Clusterin, FAS, M30, NMP22, sTNFR1, sTNFR2, tPA |
| Angiogenesis | CXCL16, HAD, IL-1α, IL-1β, IL-8, VEGF |
| Coagulation | D-dimer, IL-1β, PAI-1/tPA, TM, TNFα, VEGF |
| Differentiation | CEA, CK-20 TGF-β1, tPA |
| DNA/oxidative damage or stress | 8-OHdG |
| Growth and sustained proliferation | EGF, HAD, IL-1α, IL-1β, IL-7, IL-8, NMP22, TGF-β1, tPA |
| Glycolysis | NSE, Progranulin |
| Hormone | Prolactin, PSA/tPSA |
| Hypertension | Creatinine, Microalbumin, Midkine, Osmolality, Protein |
| Immune response | BTA, IFNɣ, IL-2, sIL-2Ra, IL-3, IL-4, IL-6, sIL-6R, IL-8, IL-10, IL-12p70, TGF-β1 |
| Inflammation | CRP, CXCL16, GRO-α, HAD, IL-6, IL-8, IL-13, IL-18, IL-23, MCP-1, Midkine, NGAL, TNFα, sTNFR1, sTNFR2 |
| Invasion/metastases | CD44, CEA, Cystatin B, HAD, IL-8, M2PK, MMP-9, MMP-9/NGAL, NGAL, S100A4, MMP-9/TIMP-1, TGF-β1 |
| Ion Transport | LASP1 |
| Nutrition/metabolism | ACR, A-FABP, Cholesterol, Cystatin C, HDL, LDL, pERK, Triglycerides |

1. **Analytical procedures**

Patient samples were run in triplicate and the results are expressed as a mean + SD (n=3). Biochip Array Technology (Randox Clinical Laboratory Services (RCLS), Antrim, Northern Ireland, UK) was used for the simultaneous detection of multiple analytes from a single patient sample (urine or serum). Samples were analysed following manufacturer’s instructions (Randox Laboratories Ltd, Crumlin, UK).

**Biochip Array Technology and Biomarker Limits of Detection**

Biochip Array Technology (BAT) was used by Randox Clinical Laboratory Services (RCLS), Randox Science Park, Antrim, UK by scientists blinded to patient data, for the simultaneous detection of multiple biomarkers from a single patient sample ^1^. The analytical sensitivity of the biochip(s) was as follows: cystatin C 0.60 ng/ml; EGF 2.5 pg/ml; IFNγ 2.1 pg/ml; IL-2 4.8 pg/ml; IL-2Ra 0.12 ng/ml; IL-23 13.0 pg/ml; IL-3 8.78 pg/ml; IL-4 6.6 pg/ml; IL-6 1.2 pg/ml; IL-6R 0.62 ng/ml; IL-7 1.11 pg/ml; IL-8 7.9 pg/ml; IL-10 1.1 pg/ml; IL-12p70 2.61 pg/ml; IL-13 5.23 pg/ml; VEGF 14.6 pg/ml; TNFα 4.4 pg/ml; IL-1α 0.8 pg/ml; IL-1β 1.6 pg/ml; MCP-1 13.2 pg/ml; NSE 0.26 ng/ml; NGAL 17.8 ng/ml; sTNFR1 0.24 ng/ml; D-dimer 2.1 ng/ml; sTNFR2 0.2 ng/ml; and CRP 0.67 mg/ml. Functional sensitivity for CEA and PSA (free and total) on the biochip were 0.29, 0.02 and 0.45 ng/ml, respectively.  All biochips were run on an Evidence Investigator analyser according to manufacturer’s instructions (Randox Laboratories Ltd, Crumlin, UK). The analytical sensitivity for HDL, LDL and cholesterol were as follows: direct HDL cholesterol (HDL) 0.189 mmol/l (7.30 mg/dl), direct LDL cholesterol (LDL) 0.189 mmol/l (7.30 mg/dl) and cholesterol 0.865 mmol/l (33.4 mg/dl), respectively.  HDL, LDL and cholesterol were run on a Daytona analyser (Randox Laboratories Ltd, Crumlin, UK).  The analytical sensitivity for urinary microalbumin was 5.11 mg/l. Microalbumin was analysed on a Daytona Plus analyser (Randox, Crumlin, UK).  The analytical sensitivity for prolactin was 6.52 mIU/l. Prolactin was run on an Evidence Evolution analyser (Randox, Crumlin, UK).  The analytical sensitivity for cystatin C was 0.4 mg/l. Serum Cystatin C was run on a Daytona analyser (RCLS, Antrim, UK). Triglycerides were run on a Daytona analyser (RCLS, Antrim, UK). Creatinine (μmol/l) measurements were performed by Randox Testing Services, Crumlin, UK, using a quantitative *in vitro* diagnostic assay from Randox (Crumlin, UK) on a Daytona analyser, according to manufacturers’ instructions (Randox). The creatinine assay is linear up to 66,000 μmol/l and has a lower sensitivity of 311 μmol/l.

**Commercial ELISA kits**

The following biomarkers were detected using commercially available ELISA kits, as per manufactures instructions; all patient samples were run in triplicate: 8-hydroxy 2 deoxyguanosine (8OHdG), MDD 0.1 ng/ml (Cell Biolabs, San Diego, US); Bladder tumour antigen (BTA), MDD 0.65 U/ml (Polymedco, New York, US); Cluster of differentiation 44 (CD44), MDD <0.113 ng/ml (Abcam, Cambridge, UK); UBC II (CK-8, CK-18), MDD 0.1 ng/ml (IDL, Bromma, Sweden); Cytokeratin-20, MDD 0.1 ng/ml (CK-20) (BlueGene, Shanghai, China); Clusterin, MDD 0.189 ng/ml (R&D Systems, Abingdon, UK); CXCL16, MDD 0.007 ng/ml (R&D Systems, Abingdon, UK); Cystatin B, MDD 0.013 ng/ml (R&D Systems, Abingdon, UK);  Epithelial growth factor (EGF), MDD 25 pg/ml (Randox, Antrim, UK); Fatty acid-binding protein – adipose (FABP-A), MDD 0.05 ng/ml (Biovendor, Abingdon, UK); Tumour necrosis factor receptor superfamily member 6 (FAS), 5 pg/ml (RayBio, Georgia, US); Hyaluronic acid (HAD), MDD 0.1 U/l (MyBioSource, San Diego, US); C-X-C ligand 1 motif/growth regulated alpha protein (CXCL1/GROα) MDD 10 pg/ml (R&D Systems, Abingdon, UK); Interleukin 18 (IL-18), MDD 42.8 pg/ml (Randox, Antrim, UK); LIM and SH3 domain (LASP-1), MDD 6.25 pg/ml (Cusabio, Houston, US); Muscle type-2 pyruvate kinase (M2-PK), MDD 3.4 ng/ml (Randox, Antrim, UK); Caspase-cleaved CK-18 fragments (M30), MDD 20 U/L (Previva, Paudex, Switzerland); Midkine, MDD 8 pg/ml (CellMid, Sydney, Australia); Matrix metallopeptidase 9/Neutrophil gelatinase-associated lipocalin complex (MMP-9/NGAL), MDD 0.013 ng/ml (R&D Systems, Abingdon, UK); Matrix metallopeptidase 9/Tissue inhibitor of metallopeptidase-1 complex (MMP-9/TIMP-1), MDD 0.0469 ng/ml (R&D Systems, Abingdon, UK); Plasminogen activator inhibitor-1/Tissue plasminogen activator complex (PAI-1/tPA), MDD 0.04 ngml (AssayPro, Missouri, US); Phospho-extracellular signal-related kinase (pERK), MDD 18.75 pg/ml (MyBioSource, San Diego, UK); Progranulin, MDD 0.17 ng/ml (R&D Systems, Abingdon, UK), S100 calcium-binding protein A4 (S100A4), MDD 0.225 ng/ml (Cusabio, Houston, US); Transforming growth factor beta-1 (TGFβ1), MDD 4.61 pg/ml (R&D Systems, Abingdon, UK); Thrombomodulin, MDD 7.82 pg/ml (R&D Systems, Abingdon, UK) and Tissue plasminogen activator (TPA), MDD 0.01 ng/ml (Abcam, Cambridge, UK).

**Point of care assays and investigations**

At recruitment, patient urine samples were collected prior to cytoscopic examination and evaluated using the point of care test (POCT) Nuclear Matrix Protein 22 (NMP22) (BladderChek, Alere, US), according to manufacturer’s instructions (MDD <10 U/ml were negative). Aution sticks 10EA, used for dipstick urinalysis, were interpreted using a PocketChem analyser (Arkray Inc, Japan).

**Osmolality**

Osmolality (mOsm) was determined using a Lӧser Micro-osmometer according to manufacturer’s instructions (Loser Messtechnik, Berlin, Germany).

**Total Urinary Protein (Bradford Assay)**

Total urinary protein levels (mg/ml) were determined, in triplicate, by Bradford assay (Pierce, Rockford, IL, USA) using a stock solution of BSA (Sigma) as standard (1 mg/ml). Patient urine samples (10 μl/patient), after centrifugation (1200 g, 10 minutes, 4^o^C), were mixed with Bradford reagent (1 ml) and allowed to stand for 5 minutes.  The samples were read on a Hitachi Spectrophotometer (Model No. U-2800) at A_595_nm. Total urinary protein was determined using a BSA calibration chart.

Data below the Limit of Detection (LOD)/Mean Detectable Dose (MDD) – when data was below the LOD/MDD for any given test, 90% of the LOD/MDD was used in the analysis.

1. **Results of logistic regression**

For logistic regression, two approaches were tested: univariate linear logistic regression and non-linear regression (natural cubic splines). ANOVA tests were performed to assess which approach better represented the relationships in the data. For biomarkers with statistically higher model performance when non-linear models were used, we presented the results of this modelling. If there was no increase in model performance when using a non-linear approach, we presented the results of the simpler model (linear logistic regression). The model used in the final modelling is indicated in the last column of the tables below.

**Table B. Logistic regression classification results for every marker in different types of samples (urine or serum) for both genders. Number of cases in the sick class: 555, number of cases in healthy class: 120. Sick class was assumed to be positive.**

| **Biomarker** | **AUC** | **Accuracy** | **Sensitivity** | **Specificity** | **Balanced**  **accuracy** | **Type of regression** |
| --- | --- | --- | --- | --- | --- | --- |
| 8-0HdG (ng/ml) (urine) | 0.596 | 0.837 | 0.994 | 0 | 0.497 |  |
| ACR (urine) | 0.502 | 0.821 | 1 | 0 | 0.5 | Natural cubic spline |
| BTA (U/ml) (urine) | 0.623 | 0.821 | 1 | 0 | 0.5 | Natural cubic spline |
| CD44 (ng/ml) (serum) | 0.513 | 0.842 | 1 | 0 | 0.5 |  |
| CEA (ng/ml) (serum) | 0.618 | 0.821 | 1 | 0 | 0.5 | Natural cubic spline |
| CK-20 (ng/ml) (urine) | 0.544 | 0.842 | 1 | 0 | 0.5 |  |
| Clusterin (ng/ml) (urine) | 0.680 | 0.837 | 0.994 | 0 | 0.497 |  |
| Creatinine (mmolL) (urine) | 0.712 | 0.842 | 0.994 | 0.031 | 0.513 |  |
| CRP (mg/ml) (urine) | 0.541 | 0.842 | 1 | 0 | 0.5 |  |
| CRP (mg/ml) (serum) | 0.499 | 0.842 | 1 | 0 | 0.5 |  |
| CXCL16 (ng/ml) (urine) | 0.749 | 0.821 | 1 | 0 | 0.5 | Natural cubic spline |
| Cystatin B (ng/ml) (urine) | 0.523 | 0.842 | 1 | 0 | 0.5 |  |
| Cystatin C (ng/ml) (urine) | 0.744 | 0.845 | 0.993 | 0.167 | 0.580 | Natural cubic spline |
| Cystatin C (ng/ml) (serum) | 0.602 | 0.821 | 1 | 0 | 0.5 | Natural cubic spline |
| D-dimer (ng/ml) (urine) | 0.618 | 0.842 | 1 | 0 | 0.5 |  |
| EGF (pg/ml) (urine) | 0.593 | 0.842 | 1 | 0 | 0.5 |  |
| EGF (pg/ml) (serum) | 0.592 | 0.842 | 1 | 0 | 0.5 |  |
| FABP-A (ng/ml) (serum) | 0.608 | 0.821 | 1 | 0 | 0.5 | Natural cubic spline |
| FAS (pg/ml) (urine) | 0.581 | 0.821 | 1 | 0 | 0.5 | Natural cubic spline |
| GROα (pg/ml) (serum) | 0.541 | 0.842 | 1 | 0 | 0.5 |  |
| HAD (U/l) (serum) | 0.605 | 0.815 | 0.993 | 0 | 0.496 | Natural cubic spline |
| IFNɣ(pg/ml) (urine) | 0.520 | 0.842 | 1 | 0 | 0.5 |  |
| IFNɣ (pg/ml) (serum) | 0.488 | 0.842 | 1 | 0 | 0.5 |  |
| IL-1α (pg/ml) (urine) | 0.561 | 0.842 | 1 | 0 | 0.5 |  |
| IL-1α (pg/ml) (serum) | 0.492 | 0.842 | 1 | 0 | 0.5 |  |
| IL-1β (pg/ml) (urine) | 0.524 | 0.842 | 1 | 0 | 0.5 |  |
| IL-1β (pg/ml) (serum) | 0.461 | 0.842 | 1 | 0 | 0.5 |  |
| IL-2 (pg/ml) (urine) | 0.515 | 0.842 | 1 | 0 | 0.5 |  |
| IL-2 (pg/ml) (serum) | 0.517 | 0.842 | 1 | 0 | 0.5 |  |
| IL-3 (pg/ml) (urine) | 0.520 | 0.842 | 1 | 0 | 0.5 |  |
| IL-4 (pg/ml) (urine) | 0.527 | 0.842 | 1 | 0 | 0.5 |  |
| IL-4 (pg/ml) (serum) | 0.510 | 0.842 | 1 | 0 | 0.5 |  |
| IL-6 (pg/ml) (urine) | 0.669 | 0.842 | 1 | 0 | 0.5 |  |
| IL-6 (pg/ml) (serum) | 0.657 | 0.842 | 1 | 0 | 0.5 |  |
| IL-7 (pg/ml) (urine) | 0.751 | 0.821 | 1 | 0 | 0.5 | Natural cubic spline |
| IL-8 (pg/ml) (urine) | 0.646 | 0.821 | 1 | 0 | 0.5 | Natural cubic spline |
| IL-8 (pg/ml) (serum) | 0.521 | 0.842 | 1 | 0 | 0.5 |  |
| IL-10 (pg/ml) (urine) | 0.485 | 0.842 | 1 | 0 | 0.5 |  |
| IL-10 (pg/ml) (serum) | 0.611 | 0.842 | 1 | 0 | 0.5 |  |
| IL-12p70 (pg/ml) (urine) | 0.477 | 0.842 | 1 | 0 | 0.5 |  |
| IL-13 (pg/ml) (urine) | 0.555 | 0.842 | 1 | 0 | 0.5 |  |
| IL-18 (pg/ml) (serum) | 0.571 | 0.842 | 1 | 0 | 0.5 |  |
| IL-23 (pg/ml) (urine) | 0.504 | 0.842 | 1 | 0 | 0.5 |  |
| LASP1 (pg/ml) (serum) | 0.550 | 0.842 | 1 | 0 | 0.5 |  |
| M30 (U/l) (serum) | 0.573 | 0.842 | 1 | 0 | 0.5 |  |
| M2PK (ng/ml) (serum) | 0.559 | 0.842 | 1 | 0 | 0.5 |  |
| MCP-1 (pg/ml) (urine) | 0.734 | 0.842 | 1 | 0 | 0.5 |  |
| MCP-1 (pg/ml) (serum) | 0.531 | 0.842 | 1 | 0 | 0.5 |  |
| Microalbumin (mg/l) (urine) | 0.746 | 0.842 | 1 | 0 | 0.5 |  |
| Midkine (pg/ml) (urine) | 0.673 | 0.842 | 0.994 | 0.031 | 0.513 |  |
| MMP-9 (ng/ml) (urine) | 0.542 | 0.821 | 1 | 0 | 0.5 | Natural cubic spline |
| MMP-9/NGAL (ng/ml) (urine) | 0.587 | 0.842 | 1 | 0 | 0.5 |  |
| MMP-9/TIMP-1 (ng/ml) (urine) | 0.580 | 0.842 | 1 | 0 | 0.5 |  |
| NGAL (ng/ml) (urine) | 0.616 | 0.842 | 1 | 0 | 0.5 |  |
| NSE (ng/ml) (urine) | 0.547 | 0.842 | 1 | 0 | 0.5 |  |
| Osmolality (mOsm) (urine) | 0.686 | 0.8214 | 1 | 0 | 0.5 | Natural cubic spline |
| PAI-1/tPA (ng/ml) (serum) | 0.630 | 0.842 | 1 | 0 | 0.5 |  |
| pERK (pg/ml) (urine) | 0.698 | 0.842 | 1 | 0 | 0.5 |  |
| Progranulin (ng/ml) (urine) | 0.724 | 0.847 | 1 | 0.031 | 0.516 |  |
| Prolactin (mlU/l) (serum) | 0.531 | 0.842 | 1 | 0 | 0.5 |  |
| Protein (mg/ml) (urine) | 0.703 | 0.842 | 1 | 0 | 0.5 |  |
| PSA/tPSA (ng/ml) (serum) | 0.776 | 0.842 | 1 | 0 | 0.5 |  |
| S100A4 (ng/ml) (serum) | 0.564 | 0.842 | 1 | 0 | 0.5 |  |
| sIL-2Ra (ng/ml) (serum) | 0.538 | 0.842 | 1 | 0 | 0.5 |  |
| sIL-6R (ng/ml) (urine) | 0.484 | 0.842 | 1 | 0 | 0.5 |  |
| TGF-β1 (pg/ml) (urine) | 0.663 | 0.821 | 1 | 0 | 0.5 | Natural cubic spline |
| TM (ng/ml) (urine) | 0.712 | 0.821 | 1 | 0 | 0.5 | Natural cubic spline |
| TNFα (pg/ml) (urine) | 0.486 | 0.842 | 1 | 0 | 0.5 |  |
| TNFα (pg/ml) (serum) | 0.358 | 0.821 | 1 | 0 | 0.5 | Natural cubic spline |
| sTNFR1 (ng/ml) (urine) | 0.743 | 0.857 | 0.988 | 0.156 | 0.572 |  |
| sTNRF2 (ng/ml) (urine) | 0.701 | 0.842 | 1 | 0 | 0.5 |  |
| tPA (ng/ml) (urine) | 0.504 | 0.842 | 1 | 0 | 0.5 |  |
| VEGF (pg/ml) (urine) | 0.742 | 0.821 | 1 | 0 | 0.5 | Natural cubic spline |
| VEGF (pg/ml) (serum) | 0.557 | 0.842 | 1 | 0 | 0.5 |  |
| HDL (mmol/l) (serum) | 0.705 | 0.842 | 1 | 0 | 0.5 |  |
| LDL (mmol/l) (serum) | 0.641 | 0.842 | 1 | 0 | 0.5 |  |
| Triglycerides (mmol/l) (serum) | 0.535 | 0.842 | 1 | 0 | 0.5 |  |
| Cholesterol (mmol/l) (serum) | 0.686 | 0.842 | 1 | 0 | 0.5 |  |
| NMP22 (POC) | 0.525 | 0.842 | 1 | 0 | 0.5 |  |

**Table C. Logistic regression classification results for every marker in different types of samples (urine or serum) for female.** **Number of cases in the sick class: 111, number of cases in healthy class: 79. Sick class was assumed to be positive.**

| **Biomarker** | **AUC** | **Accuracy** | **Sensitivity** | **Specificity** | **Balanced**  **accuracy** | **Type of regression** |
| --- | --- | --- | --- | --- | --- | --- |
| 8-0HdG (ng/ml) (urine) | 0.656 | 0.667 | 0.8 | 0.455 | 0.627 |  |
| ACR (urine) | 0.664 | 0.667 | 1 | 0.136 | 0.568 |  |
| BTA (U/ml) (urine) | 0.608 | 0.609 | 0.852 | 0.263 | 0.558 | Natural cubic spline |
| CD44 (ng/ml) (serum) | 0.525 | 0.596 | 0.914 | 0.091 | 0.503 |  |
| CEA (ng/ml) (serum) | 0.570 | 0.630 | 0.852 | 0.316 | 0.584 | Natural cubic spline |
| CK-20 (ng/ml) (urine) | 0.488 | 0.614 | 1 | 0 | 0.5 |  |
| Clusterin (ng/ml) (urine) | 0.668 | 0.614 | 0.743 | 0.409 | 0.576 |  |
| Creatinine (mmolL) (urine) | 0.655 | 0.632 | 0.743 | 0.455 | 0.599 |  |
| CRP (mg/ml) (urine) | 0.589 | 0.614 | 1 | 0 | 0.5 |  |
| CRP (mg/ml) (serum) | 0.542 | 0.579 | 0.8 | 0.227 | 0.514 |  |
| CXCL16 (ng/ml) (urine) | 0.774 | 0.684 | 0.743 | 0.591 | 0.667 |  |
| Cystatin B (ng/ml) (urine) | 0.471 | 0.649 | 0.886 | 0.273 | 0.579 |  |
| Cystatin C (ng/ml) (urine) | 0.638 | 0.596 | 0.657 | 0.5 | 0.579 |  |
| Cystatin C (ng/ml) (serum) | 0.602 | 0.587 | 0.778 | 0.316 | 0.547 | Natural cubic spline |
| D-dimer (ng/ml) (urine) | 0.537 | 0.491 | 0.4 | 0.636 | 0.518 |  |
| EGF (pg/ml) (urine) | 0.585 | 0.614 | 0.771 | 0.364 | 0.568 |  |
| EGF (pg/ml) (serum) | 0.565 | 0.561 | 0.714 | 0.318 | 0.516 |  |
| FABP-A (ng/ml) (serum) | 0.591 | 0.614 | 1 | 0 | 0.5 |  |
| FAS (pg/ml) (urine) | 0.518 | 0.526 | 0.771 | 0.136 | 0.454 |  |
| GROα (pg/ml) (serum) | 0.508 | 0.544 | 0.686 | 0.318 | 0.502 |  |
| HAD (U/l) (serum) | 0.590 | 0.614 | 1 | 0 | 0.5 |  |
| IFNɣ(pg/ml) (urine) | 0.543 | 0.614 | 1 | 0 | 0.5 |  |
| IFNɣ (pg/ml) (serum) | 0.463 | 0.561 | 0.914 | 0.000 | 0.457 |  |
| IL-1α (pg/ml) (urine) | 0.578 | 0.561 | 0.657 | 0.409 | 0.533 |  |
| IL-1α (pg/ml) (serum) | 0.492 | 0.614 | 1 | 0 | 0.5 |  |
| IL-1β (pg/ml) (urine) | 0.606 | 0.596 | 0.6 | 0.591 | 0.595 |  |
| IL-1β (pg/ml) (serum) | 0.534 | 0.614 | 1 | 0 | 0.5 |  |
| IL-2 (pg/ml) (urine) | 0.543 | 0.614 | 1 | 0 | 0.5 |  |
| IL-2 (pg/ml) (serum) | 0.468 | 0.614 | 1 | 0 | 0.5 |  |
| IL-3 (pg/ml) (urine) | 0.514 | 0.614 | 1 | 0 | 0.5 |  |
| IL-4 (pg/ml) (urine) | 0.574 | 0.614 | 1 | 0 | 0.5 |  |
| IL-4 (pg/ml) (serum) | 0.490 | 0.596 | 0.914 | 0.091 | 0.503 |  |
| IL-6 (pg/ml) (urine) | 0.590 | 0.579 | 0.6 | 0.545 | 0.573 |  |
| IL-6 (pg/ml) (serum) | 0.458 | 0.614 | 0.857 | 0.227 | 0.542 |  |
| IL-7 (pg/ml) (urine) | 0.623 | 0.614 | 0.629 | 0.591 | 0.610 |  |
| IL-8 (pg/ml) (urine) | 0.708 | 0.652 | 0.889 | 0.316 | 0.602 | Natural cubic spline |
| IL-8 (pg/ml) (serum) | 0.554 | 0.632 | 1 | 0.045 | 0.523 |  |
| IL-10 (pg/ml) (urine) | 0.521 | 0.614 | 1 | 0 | 0.5 |  |
| IL-10 (pg/ml) (serum) | 0.512 | 0.614 | 1 | 0 | 0.5 |  |
| IL-12p70 (pg/ml) (urine) | 0.618 | 0.614 | 0.914 | 0.136 | 0.525 |  |
| IL-13 (pg/ml) (urine) | 0.604 | 0.587 | 1 | 0 | 0.5 | Natural cubic spline |
| IL-18 (pg/ml) (serum) | 0.399 | 0.474 | 0.743 | 0.045 | 0.394 |  |
| IL-23 (pg/ml) (urine) | 0.486 | 0.596 | 0.971 | 0 | 0.486 |  |
| LASP1 (pg/ml) (serum) | 0.513 | 0.614 | 1 | 0 | 0.5 |  |
| M30 (U/l) (serum) | 0.519 | 0.614 | 1 | 0 | 0.5 |  |
| M2PK (ng/ml) (serum) | 0.508 | 0.614 | 1 | 0 | 0.5 |  |
| MCP-1 (pg/ml) (urine) | 0.599 | 0.596 | 0.657 | 0.5 | 0.579 |  |
| MCP-1 (pg/ml) (serum) | 0.523 | 0.561 | 0.857 | 0.091 | 0.474 |  |
| Microalbumin (mg/l) (urine) | 0.734 | 0.649 | 0.571 | 0.773 | 0.672 |  |
| Midkine (pg/ml) (urine) | 0.663 | 0.630 | 0.889 | 0.263 | 0.576 | Natural cubic spline |
| MMP-9 (ng/ml) (urine) | 0.526 | 0.614 | 1 | 0 | 0.5 |  |
| MMP-9/NGAL (ng/ml) (urine) | 0.582 | 0.684 | 0.857 | 0.409 | 0.633 |  |
| MMP-9/TIMP-1 (ng/ml) (urine) | 0.597 | 0.614 | 1 | 0 | 0.5 |  |
| NGAL (ng/ml) (urine) | 0.526 | 0.509 | 0.686 | 0.227 | 0.456 |  |
| NSE (ng/ml) (urine) | 0.642 | 0.587 | 1 | 0 | 0.5 | Natural cubic spline |
| Osmolality (mOsm) (urine) | 0.591 | 0.684 | 0.857 | 0.409 | 0.633 |  |
| PAI-1/tPA (ng/ml) (serum) | 0.602 | 0.632 | 0.857 | 0.273 | 0.565 |  |
| pERK (pg/ml) (urine) | 0.726 | 0.702 | 0.8 | 0.545 | 0.673 |  |
| Progranulin (ng/ml) (urine) | 0.646 | 0.596 | 0.714 | 0.409 | 0.562 |  |
| Prolactin (mlU/l) (serum) | 0.586 | 0.614 | 1 | 0 | 0.5 |  |
| Protein (mg/ml) (urine) | 0.750 | 0.609 | 0.889 | 0.211 | 0.550 | Natural cubic spline |
| PSA/tPSA (ng/ml) (serum) | 0.690 | 0.614 | 1 | 0 | 0.5 |  |
| S100A4 (ng/ml) (serum) | 0.470 | 0.579 | 0.857 | 0.136 | 0.497 |  |
| sIL-2Ra (ng/ml) (serum) | 0.471 | 0.614 | 1 | 0 | 0.5 |  |
| sIL-6R (ng/ml) (urine) | 0.500 | 0.614 | 1 | 0 | 0.5 |  |
| TGF-β1 (pg/ml) (urine) | 0.512 | 0.587 | 0.815 | 0.263 | 0.539 | Natural cubic spline |
| TM (ng/ml) (urine) | 0.647 | 0.596 | 0.686 | 0.455 | 0.570 |  |
| TNFα (pg/ml) (urine) | 0.551 | 0.614 | 1 | 0 | 0.5 |  |
| TNFα (pg/ml) (serum) | 0.470 | 0.609 | 1 | 0.053 | 0.526 | Natural cubic spline |
| sTNFR1 (ng/ml) (urine) | 0.658 | 0.674 | 0.815 | 0.474 | 0.644 | Natural cubic spline |
| sTNRF2 (ng/ml) (urine) | 0.609 | 0.544 | 0.543 | 0.545 | 0.544 |  |
| tPA (ng/ml) (urine) | 0.575 | 0.596 | 0.857 | 0.182 | 0.519 |  |
| VEGF (pg/ml) (urine) | 0.769 | 0.702 | 0.971 | 0.273 | 0.622 |  |
| VEGF (pg/ml) (serum) | 0.583 | 0.544 | 0.743 | 0.227 | 0.485 |  |
| HDL (mmol/l) (serum) | 0.480 | 0.561 | 0.771 | 0.227 | 0.499 |  |
| LDL (mmol/l) (serum) | 0.536 | 0.474 | 0.771 | 0 | 0.386 |  |
| Triglycerides (mmol/l) (serum) | 0.557 | 0.544 | 0.8 | 0.136 | 0.468 |  |
| Cholesterol (mmol/l) (serum) | 0.519 | 0.509 | 0.686 | 0.227 | 0.456 |  |
| NMP22 (POC) | 0.520 | 0.614 | 1 | 0 | 0.5 |  |

**Table D. Logistic regression classification results for every marker in different types of samples (urine or serum) for male.** **Number of cases in the sick class: 441, number of cases in healthy class: 44. Sick class was assumed to be positive.**

| **Biomarker** | **AUC** | **Accuracy** | **Sensitivity** | **Specificity** | **Balanced**  **accuracy** | **Type of regression** |
| --- | --- | --- | --- | --- | --- | --- |
| 8-0HdG (ng/ml) (urine) | 0.695 | 0.910 | 1 | 0 | 0.5 |  |
| ACR (urine) | 0.599 | 0.910 | 1 | 0 | 0.5 |  |
| BTA (U/ml) (urine) | 0.674 | 0.910 | 1 | 0 | 0.5 |  |
| CD44 (ng/ml) (serum) | 0.578 | 0.910 | 1 | 0 | 0.5 |  |
| CEA (ng/ml) (serum) | 0.597 | 0.910 | 1 | 0 | 0.5 |  |
| CK-20 (ng/ml) (urine) | 0.547 | 0.910 | 1 | 0 | 0.5 |  |
| Clusterin (ng/ml) (urine) | 0.724 | 0.910 | 1 | 0 | 0.5 |  |
| Creatinine (mmolL) (urine) | 0.676 | 0.910 | 1 | 0 | 0.5 |  |
| CRP (mg/ml) (urine) | 0.586 | 0.910 | 1 | 0 | 0.5 |  |
| CRP (mg/ml) (serum) | 0.547 | 0.910 | 1 | 0 | 0.5 |  |
| CXCL16 (ng/ml) (urine) | 0.679 | 0.917 | 1 | 0 | 0.5 | Natural cubic spline |
| Cystatin B (ng/ml) (urine) | 0.698 | 0.910 | 1 | 0 | 0.5 |  |
| Cystatin C (ng/ml) (urine) | 0.695 | 0.910 | 1 | 0 | 0.5 |  |
| Cystatin C (ng/ml) (serum) | 0.764 | 0.910 | 1 | 0 | 0.5 |  |
| D-dimer (ng/ml) (urine) | 0.696 | 0.910 | 1 | 0 | 0.5 |  |
| EGF (pg/ml) (urine) | 0.775 | 0.917 | 1 | 0 | 0.5 | Natural cubic spline |
| EGF (pg/ml) (serum) | 0.540 | 0.910 | 1 | 0 | 0.5 |  |
| FABP-A (ng/ml) (serum) | 0.598 | 0.910 | 1 | 0 | 0.5 |  |
| FAS (pg/ml) (urine) | 0.579 | 0.917 | 1 | 0 | 0.5 | Natural cubic spline |
| GROα (pg/ml) (serum) | 0.597 | 0.910 | 1 | 0 | 0.5 |  |
| HAD (U/l) (serum) | 0.691 | 0.917 | 1 | 0 | 0.5 | Natural cubic spline |
| IFNɣ(pg/ml) (urine) | 0.515 | 0.910 | 1 | 0 | 0.5 |  |
| IFNɣ (pg/ml) (serum) | 0.511 | 0.910 | 1 | 0 | 0.5 |  |
| IL-1α (pg/ml) (urine) | 0.731 | 0.910 | 1 | 0 | 0.5 |  |
| IL-1α (pg/ml) (serum) | 0.486 | 0.910 | 1 | 0 | 0.5 |  |
| IL-1β (pg/ml) (urine) | 0.625 | 0.910 | 1 | 0 | 0.5 |  |
| IL-1β (pg/ml) (serum) | 0.512 | 0.910 | 1 | 0 | 0.5 |  |
| IL-2 (pg/ml) (urine) | 0.527 | 0.910 | 1 | 0 | 0.5 |  |
| IL-2 (pg/ml) (serum) | 0.495 | 0.910 | 1 | 0 | 0.5 |  |
| IL-3 (pg/ml) (urine) | 0.519 | 0.910 | 1 | 0 | 0.5 |  |
| IL-4 (pg/ml) (urine) | 0.550 | 0.910 | 1 | 0 | 0.5 |  |
| IL-4 (pg/ml) (serum) | 0.516 | 0.910 | 1 | 0 | 0.5 |  |
| IL-6 (pg/ml) (urine) | 0.761 | 0.910 | 1 | 0 | 0.5 |  |
| IL-6 (pg/ml) (serum) | 0.613 | 0.910 | 1 | 0 | 0.5 |  |
| IL-7 (pg/ml) (urine) | 0.635 | 0.910 | 1 | 0 | 0.5 |  |
| IL-8 (pg/ml) (urine) | 0.708 | 0.910 | 1 | 0 | 0.5 |  |
| IL-8 (pg/ml) (serum) | 0.632 | 0.910 | 1 | 0 | 0.5 |  |
| IL-10 (pg/ml) (urine) | 0.538 | 0.910 | 1 | 0 | 0.5 |  |
| IL-10 (pg/ml) (serum) | 0.547 | 0.903 | 0.992 | 0 | 0.496 |  |
| IL-12p70 (pg/ml) (urine) | 0.441 | 0.910 | 1 | 0 | 0.5 |  |
| IL-13 (pg/ml) (urine) | 0.643 | 0.917 | 1 | 0 | 0.5 | Natural cubic spline |
| IL-18 (pg/ml) (serum) | 0.600 | 0.910 | 1 | 0 | 0.5 |  |
| IL-23 (pg/ml) (urine) | 0.476 | 0.910 | 1 | 0 | 0.5 |  |
| LASP1 (pg/ml) (serum) | 0.506 | 0.910 | 1 | 0 | 0.5 |  |
| M30 (U/l) (serum) | 0.487 | 0.910 | 1 | 0 | 0.5 |  |
| M2PK (ng/ml) (serum) | 0.540 | 0.910 | 1 | 0 | 0.5 |  |
| MCP-1 (pg/ml) (urine) | 0.752 | 0.910 | 1 | 0 | 0.5 |  |
| MCP-1 (pg/ml) (serum) | 0.462 | 0.910 | 1 | 0 | 0.5 |  |
| Microalbumin (mg/l) (urine) | 0.721 | 0.910 | 1 | 0 | 0.5 |  |
| Midkine (pg/ml) (urine) | 0.570 | 0.910 | 1 | 0 | 0.5 |  |
| MMP-9 (ng/ml) (urine) | 0.506 | 0.910 | 1 | 0 | 0.5 |  |
| MMP-9/NGAL (ng/ml) (urine) | 0.655 | 0.910 | 1 | 0 | 0.5 |  |
| MMP-9/TIMP-1 (ng/ml) (urine) | 0.571 | 0.910 | 1 | 0 | 0.5 |  |
| NGAL (ng/ml) (urine) | 0.654 | 0.910 | 1 | 0 | 0.5 |  |
| NSE (ng/ml) (urine) | 0.604 | 0.910 | 1 | 0 | 0.5 |  |
| Osmolality (mOsm) (urine) | 0.603 | 0.917 | 1 | 0 | 0.5 | Natural cubic spline |
| PAI-1/tPA (ng/ml) (serum) | 0.614 | 0.910 | 1 | 0 | 0.5 |  |
| pERK (pg/ml) (urine) | 0.581 | 0.910 | 1 | 0 | 0.5 |  |
| Progranulin (ng/ml) (urine) | 0.734 | 0.910 | 1 | 0 | 0.5 |  |
| Prolactin (mlU/l) (serum) | 0.523 | 0.910 | 1 | 0 | 0.5 |  |
| Protein (mg/ml) (urine) | 0.647 | 0.910 | 1 | 0 | 0.5 |  |
| PSA/tPSA (ng/ml) (serum) | 0.676 | 0.910 | 1 | 0 | 0.5 |  |
| S100A4 (ng/ml) (serum) | 0.690 | 0.910 | 1 | 0 | 0.5 |  |
| sIL-2Ra (ng/ml) (serum) | 0.576 | 0.910 | 1 | 0 | 0.5 |  |
| sIL-6R (ng/ml) (urine) | 0.466 | 0.910 | 1 | 0 | 0.5 |  |
| TGF-β1 (pg/ml) (urine) | 0.668 | 0.917 | 1 | 0 | 0.5 | Natural cubic spline |
| TM (ng/ml) (urine) | 0.683 | 0.910 | 1 | 0 | 0.5 |  |
| TNFα (pg/ml) (urine) | 0.490 | 0.910 | 1 | 0 | 0.5 |  |
| TNFα (pg/ml) (serum) | 0.605 | 0.910 | 1 | 0 | 0.5 |  |
| sTNFR1 (ng/ml) (urine) | 0.801 | 0.910 | 1 | 0 | 0.5 |  |
| sTNRF2 (ng/ml) (urine) | 0.834 | 0.910 | 1 | 0 | 0.5 |  |
| tPA (ng/ml) (urine) | 0.532 | 0.910 | 1 | 0 | 0.5 |  |
| VEGF (pg/ml) (urine) | 0.624 | 0.910 | 1 | 0 | 0.5 |  |
| VEGF (pg/ml) (serum) | 0.497 | 0.910 | 1 | 0 | 0.5 |  |
| HDL (mmol/l) (serum) | 0.653 | 0.917 | 1 | 0 | 0.5 | Natural cubic spline |
| LDL (mmol/l) (serum) | 0.658 | 0.910 | 1 | 0 | 0.5 |  |
| Triglycerides (mmol/l) (serum) | 0.582 | 0.910 | 1 | 0 | 0.5 |  |
| Cholesterol (mmol/l) (serum) | 0.663 | 0.910 | 1 | 0 | 0.5 |  |
| NMP22 (POC) | 0.518 | 0.910 | 1 | 0 | 0.5 |  |

**Table E. Comparison of the decision boundaries for each algorithm. The cut-off values are shown as raw measurement results. The unit of the values presented goes as follows: BTA (U/ml), serum_CD44 (ng/ml) , serum_CEA (ng/ml), Clusterin (ng/ml), Creatinine (mmolL), serum_CRP (mg/ml), CXCL16 (ng/ml), Cystatin B (ng/ml), Cystatin C (ng/ml), serum_Cystatin C (ng/ml), D-dimer (ng/ml), EGF (pg/ml, serum_ HAD (U/l), serum_ IL-4 (pg/ml), IL-7 (pg/ml), IL-8 (pg/ml), MCP-1 (pg/ml, Microalbumin (mg/l), Midkine (pg/ml), NGAL (ng/ml),Osmolality (mOsm), pERK (pg/ml),Progranulin (ng/ml), serum_tPSA (ng/ml), Protein (mg/ml),serum_S100A4 (ng/ml), TGF-β1 (pg/ml), sTNFR1 (ng/ml), serum_ VEGF (pg/ml), serum_ HDL (mmol/l).**

| Marker | CACTUS | | | Random Forest (LIME) | | | Decision Tree | | | |
| --- | --- | --- | --- | --- | --- | --- | --- | --- | --- | --- |
|  | **Both** | **Male** | **Female** | **Both** | **Male** | **Female** | **Both** | **Male** | **Female** |  |
| ACR | - | 1.74 | - | - | - | [0.32,1.02] ∨ (1.02,1.59] ∨ (1.59,3.30] ∨ (3.30,49.37] | 1.31  1.82 | 1.77 | 1.31  1.82 |  |
| Age | - | 63 | - | [40,60] ∨ (60,67] ∨ (67,73] ∨ (73,85] | [41,61] ∨ (61,68] ∨ (68,73] ∨ (74,85] | - | 61.5 | 61.5 | - |  |
| BTA | - | 9.92 | - |  |  |  |  |  |  |  |
| Clusterin |  |  |  | - | [1.4, 102.33] ∨ (102.33, 208.93] ∨ (208.93, 363.08] ∨ (363.08,2000] | - |  |  |  |  |
| Creatinine | - | - | 48.39 |  |  |  |  |  |  |  |
| Cystatin B |  |  |  | [0.7, 0.84] ∨ (0.84, 10.56] ∨ (10.56, 100] |  | - |  |  |  |  |
| Cystatin C | - | - | - | [0.54, 6.84] ∨ (6.84,20.04] ∨ (20.04, 43.45] ∨ (43.45,440] | [0.54, 20.99] ∨ (20.99, 47.86] ∨ (47.86, 440] | [0.54, 3.16] ∨ (3.16, 12.30] ∨ (12.30, 33.11] ∨ (33.11, 237.5] | 1.20 | 1.20 | 0.83 |  |
| CXCL16 | 0.03 | - | 0.02 | [0.006, 0.02] ∨ (0.02, 0.04] ∨ (0.04, 0.06] ∨ (0.06,6.878] | - | 0.006, 0.01] ∨ (0.01, 0.03] ∨ (0.03, 0.05] ∨ (0.05,1.07] |  |  |  |  |
| D-dimer | - | 16.03 | - |  |  |  |  |  |  |  |
| Gender | M | - | - | F, M | - | - | M | - | - |  |
| HAD | - | - | - | - | [0.09, 0.10] ∨ (0.10, 0.16] ∨ (0.16, 0.27] ∨ (0.27,10] | - | - | 0.32 | - |  |
| Haematuria |  |  |  |  |  |  | micro | - | micro |  |
| IL1a | - | - | 2.26 |  |  |  |  |  |  |  |
| IL7 | 1.59 | - | - |  |  |  |  |  |  |  |
| IL8 | - | - | 68.12 | - | - | [2.07, 20.89] ∨ (20.89, 77.62] ∨ (77.62, 380.19] ∨ (380.19,1821] |  |  |  |  |
| MCP_1 | 46.35 | 47.33 | - |  |  |  |  |  |  |  |
| Microalbumin | 7.87 | 13.43 | 7.87 | [4.6, 10.23] ∨ (10.23, 41.69] ∨ (41.69, 234] | - | [4.6, 5.38] ∨ (5.38, 15.70] ∨ (15.70, 234] | 7.90 | - | 7.90 |  |
| Midkine | 144.76 | - | 68.12 |  |  |  |  |  |  |  |
| NGAL | - | - | - | - | [19.29, 125.89] ∨ (125.89, 208.92] ∨ (208.92, 436.52] ∨ (436.52,2338] | - |  |  |  |  |
| pERK | - | - | 224.38 | [16.88, 186.21] ∨ (186.21, 288.40] ∨ (288.40, 501.19] ∨ (501.19, 1991.9] | - | [16.88, 138.04] ∨ (138.04, 275.42] ∨ (275.42, 457.0] ∨ (457.0, 1991.9] |  | 475.75 | - |  |
| Osmolarity | - | - | - | [83.67, 363.08] ∨ (363.08, 562.34] ∨ (562.34, 724.44] ∨ (724.44, 1065] | [83.67, 407.38] ∨ (407.38, 588.84] ∨ (588.84, 741.31] ∨ (741.31, 1065] | - |  |  |  |  |
| Progranulin | 6.72 | - | 6.72 | - | [1.15, 8.09] ∨ (8.09, 12.94] ∨ (12.94, 19.91] ∨ (19.91, 200] | [0.78, 4.69] ∨ (4.69, 16.11] ∨ (16.11, 9.78] |  |  |  |  |
| serum_CD44 |  |  |  |  |  |  | 65.22 | 65.22 | - |  |
| serum_CEA | - |  |  | - | - | [0.33, 0.90] ∨ (0.90, 1.65] ∨ (1.65, 2.82] ∨ (2.82, 12.1] |  |  |  |  |
| serum_CRP | - |  |  |  |  |  | 1.33 | - | 1.33 |  |
| serum_Cystatin C | - | 0.98 | - |  |  |  | 0.84 | - | - |  |
| serum_EGF | - | - | - | - | - | [2.25, 5.58] ∨ (5.58, 12.36] ∨ (12.36, 30.20] ∨ (30.20, 147.21] | 23.05 | - | 23.05 |  |
| serum_HDL | - |  | - | - | [0.51, 0.92] ∨ (0.92, 1.09] ∨ (1.09, 1.30] ∨ (1.30, 2.5] | - |  |  |  |  |
| serum_IL4 |  |  |  |  |  |  | 3.36 | 3.36 | - |  |
| serum_PAI_1/tPA | - | - | 6.23 | - | - | [0.73, 4.52] ∨ (4.52, 6.67] ∨ (6.67, 10.47] ∨ (10.47, 40] |  |  |  |  |
| serum_S100A4 | - |  | - | - | [0.23, 28.18] ∨ (28.18, 46.77] ∨ (46.77, 72.44] ∨ (72.44, 335.66] | - | 63.75 | - | 63.75 |  |
| serum_tPSA | 0.37 | 1.03 | - | [0.04, 0.07] ∨ (0.07, 0.80] ∨ (0.80, 2.63] ∨ (2.63, 31.25] |  | - |  |  |  |  |
| serum_VEGF | - |  | - |  |  |  | 221.68 | - | 221.68 |  |
| sTNFRI | 0.3 | - | 0.29 | [0.08, 0.7] ∨ (0.7, 1.16] ∨ (1.16, 9.98] | [0.08, 0.46] ∨ (0.46, 0.81] ∨ (0.81, 1.28] ∨ (1.28, 9.98] | - |  |  |  |  |
| sTNFRII | - | 0.34 | - |  |  |  |  |  |  |  |
| TGFb1 |  |  |  |  |  |  | 26.78 | 26.78 | - |  |
| Urinary Protein | 0.104 | 0.103 | - |  |  |  |  |  |  |  |
